# Epidemiology of eating disorders: population, prevalence, disease burden and quality of life informing public policy in Australia—a rapid review

**DOI:** 10.1186/s40337-023-00738-7

**Published:** 2023-02-15

**Authors:** Phillipa Hay, Phillip Aouad, Anvi Le, Peta Marks, Danielle Maloney, National Eating Disorder Research Consortium, Sarah Barakat, Robert Boakes, Leah Brennan, Emma Bryant, Susan Byrne, Belinda Caldwell, Shannon Calvert, Bronny Carroll, David Castle, Ian Caterson, Belinda Chelius, Lyn Chiem, Simon Clarke, Janet Conti, Lexi Crouch, Genevieve Dammery, Natasha Dzajkovski, Jasmine Fardouly, John Feneley, Nasim Foroughi, Mathew Fuller-Tyszkiewicz, Anthea Fursland, Veronica Gonzalez-Arce, Bethanie Gouldthorp, Kelly Griffin, Scott Griffiths, Ashlea Hambleton, Amy Hannigan, Mel Hart, Susan Hart, Ian Hickie, Francis Kay-Lambkin, Ross King, Michael Kohn, Eyza Koreshe, Isabel Krug, Jake Linardon, Randall Long, Amanda Long, Sloane Madden, Siân McLean, Thy Meddick, Jane Miskovic-Wheatley, Deborah Mitchison, Richard O’Kearney, Roger Paterson, Susan Paxton, Melissa Pehlivan, Genevieve Pepin, Andrea Phillipou, Judith Piccone, Rebecca Pinkus, Bronwyn Raykos, Paul Rhodes, Elizabeth Rieger, Karen Rockett, Sarah Rodan, Janice Russell, Haley Russell, Fiona Salter, Susan Sawyer, Beth Shelton, Urvashnee Singh, Sophie Smith, Evelyn Smith, Karen Spielman, Sarah Squire, Juliette Thomson, Marika Tiggemann, Ranjani Utpala, Lenny Vartanian, Andrew Wallis, Warren Ward, Sarah Wells, Eleanor Wertheim, Simon Wilksch, Michelle Williams, Stephen Touyz, Sarah Maguire

**Affiliations:** 1 Translational Health Research Institute, Western Sydney University, Sydney, NSW Australia; 2 InsideOut Institute for Eating Disorders, University of Sydney, Sydney Local Health District, Sydney, Australia; 3 Western Sydney University, Sydney, NSW Australia; 4 Healthcare Management Advisors, Robinson, VIC Australia; 5 Sydney Local Health District, New South Wales Health, Sydney, NSW Australia; 6 South West Sydney Local Health District, New South Wales Health, Sydney, NSW Australia

**Keywords:** Epidemiology, Prevalence, Incidence, Burden of disease, Eating disorders

## Abstract

**Background:**

Understanding of the epidemiology and health burden of eating disorders has progressed significantly in the last 2 decades. It was considered one of seven key areas to inform the Australian Government commissioned National Eating Disorder Research and Translation Strategy 2021–2031, as emerging research had highlighted a rise in eating disorder prevalence and worsening burden-of-illness. The aim of this review was to better understand the global epidemiology and impact of eating disorders to inform policy decision-making.

**Methods:**

Using a systematic Rapid Review methodology, ScienceDirect, PubMed and Medline (Ovid) were searched for peer-reviewed studies published between 2009 and 2021. Clear inclusion criteria were developed in consultation with experts in the field. Purposive sampling of literature was conducted, which predominately focused on higher-level evidence (meta-analyses, systematic reviews, and large epidemiological studies), synthesised, and narratively analysed.

**Results:**

135 studies were deemed eligible for inclusion in this review (N = 1324). Prevalence estimates varied. Global Lifetime prevalence of any eating disorder ranged from 0.74 to 2.2% in males, and 2.58–8.4% in females. Australian 3-month point-prevalence of broadly defined disorders was around 16% in females. Eating disorders appeared more prevalent in young people and adolescents, particularly females (in Australia: eating disorders ~ 22.2%; disordered eating ~ 25.7%). Limited evidence was found on sex, sexuality and gender diverse (LGBTQI +) individuals, particularly males, who had a six-fold increase in prevalence compared to the general male population, with increased illness impact. Similarly, limited evidence on First Australian’s (Aboriginal and Torres Strait Islander) suggests prevalence rates similar to non-Indigenous Australians. No prevalence studies were identified specifically assessing culturally and linguistically diverse populations. Global disease burden of any eating disorder was 43.4 age-standardised disability-adjusted-life-years per 100,000; increasing by 9.4% between 2007 and 2017. Australian’s total economic cost was estimated at $84 billion from years-of-life lost due to disability and death, and annual lost earnings ~ $1.646 billion.”

**Conclusions:**

There is no doubt that eating disorder prevalence and impact are on the rise, particularly in at-risk and understudied populations. Much of the evidence came from female-only samples, and Western, high-income countries which more readily have access to specialised services. Future research should examine more representative samples. There is an urgent need for more refined epidemiological methods to better understand these complex illnesses over time, to guide health policy and development-of-care.

**Supplementary Information:**

The online version contains supplementary material available at 10.1186/s40337-023-00738-7.

## Plain English summary

Our understanding of the prevalence and impact of eating disorders has improved significantly over the past 20-years. Research highlights that rates of eating disorders are increasing. To inform the development of the Eating Disorder Research and Translation Strategy 2021–2031 this review aimed to better understand the global change in prevalence and impact of eating disorders to inform policy decision-making.

Three scholarly databases were systematically searched for related research published between 2009 and 2021. Searches identified 135 studies which met our inclusion criteria. Estimates in lifetime eating disorder prevalence varied from 2.58 to 8.4% in women and girls. Findings indicated that eating disorders appeared more prevalent in young people and adolescents, particularly young women, while sexuality diverse (LGBTQI +) individuals were six-times more likely to have an eating disorder compared to the general male population. The little research suggests moderate to high prevalence of eating disorders in First Australian peoples, Australia’s spending on eating disorders was estimated at ~ $84 billion due to disability or death. There is no doubt that eating disorder prevalence and impact are on the rise. Future research should include more diverse populations to increase estimate accuracy and improve care for all.

**Supplementary Information:**

The online version contains supplementary material available at 10.1186/s40337-023-00738-7.

## Introduction

The epidemiology of eating disorders (EDs) has advanced in recent years to encompass both the ‘core’ well-specified EDs, namely Anorexia Nervosa (AN; ICD-11 Code: 6B80), Bulimia Nervosa (BN; ICD-11 Code: 6B81) and Binge Eating Disorder (BED; ICD-11 Code: 6B82) but also the spectrum of Other Specified (ICD-11 Code: 6B8Y) and Unspecified (ICD-11 Code: 6B8Z) Feeding and Eating Disorders (OSFED and UFED) and Avoidant Restrictive Food Intake Disorder or ARFID (ICD-11 Code: 6B83) [1]. Nevertheless, AN, BN and BED continue to have the largest evidence base and are commonly reported together in prevalence studies. BED and ARFID were only introduced as standalone disorders in the 2013 fifth edition of the Diagnostic and Statistical Manual of Mental Disorders [1, 2]. Prior to the DSM-5, BED (ICD-11 Code: 6B82) was described as a subtype of Eating Disorder Not Otherwise Specified (EDNOS) [1, 3]. Other Specified Feeding and Eating Disorders (OSFED; ICD-11 Code: 6B8Y) as defined in the DSM-5 include Atypical AN (A-AN); Subthreshold BN (S-BN); Subthreshold BED (S-BED); Night Eating Syndrome (NES), and Purging disorder (PD) [1].

The understanding of the population distribution and community burden of EDs has shifted notably in the last 2 decades. No longer can it be said that EDs are a problem only for young women from the developed world, a perception dating from the times of Bruch, who wrote that anorexia nervosa (AN) “…* affects young and healthy girls who have been raised in privileged, even luxurious circumstances*” [4]. There is a growing body of evidence that EDs and their related behaviours are prevalent amongst peoples from lower-income groups, non-Western cultures, and of diverse gender [5, 6, 7, 8]. Alongside this is research indicating a rise in prevalence and global burden of EDs [9]. In consideration of this, the prevalence and burden of EDs was considered one of seven key areas to inform the Australian Federal Government’s commissioning of The Australian Eating Disorder Research and Translation Strategy (AEDRTS) that aimed to identify strategic priorities and targets for building research capacity and outputs in Australia [10].

EDs are often chronic in nature and typically have an early age of onset with periods of recovery and relapse across the lifespan [11, 12]. There is substantial evidence that almost all first-time cases of well-specified EDs occur before the ages of 20 to 30 [11, 13, 14]. Therefore, the measured prevalence rates between age groups vary significantly. The highest prevalence rates are observed in children and adolescents. However, there is emerging evidence that prevalence of well-specified EDs is increasing among older adults [15].

The present paper is one of a series of Rapid Reviews, with the focus of the current paper on the epidemiology of EDs, specifically their prevalence and incidence, sociodemographic and ethnic distribution, and disease burden and impact on quality of life. The rapid reviews featured in this series, were conducted to guide the AEDRTS, and were completed over 2019–2021, in parallel and synergy with a multi-layered, multi-phased nation-wide co-designed strategy development process. Thus, the current paper aims to better understand the global epidemiology and impact of eating disorders to inform policy decision-making.

## Methods

The Australian Government funded the InsideOut Institute for Eating Disorders (IOI) to develop the AEDRTS 2021–2031 [16] in partnership with state and national stakeholders including clinicians, service providers, researchers, and experts by lived experience (encompassing consumers and families/carers). Developed through a 2-year national consultation and collaboration process, the strategy provides a roadmap to establishing EDs as national research priority and is the first disorder-specific strategy to be developed in consultation with the National Mental Health Commission. To inform the strategy, IOI commissioned Healthcare Management Advisors (HMA) to conduct a series of Rapid Reviews (RRs) to assess the current research base across the full spectrum of EDs; including knowledge gaps in ED (1) epidemiology; (2) risk factors; (3) comorbidities and medical complications; (4) screening and diagnosis; (5) prevention and early intervention; (6) psychotherapies; (7) models of care; (8) pharmacotherapies and (9) outcomes. The current paper presents the findings related to the epidemiology of EDs specifically on population trends and incidence, prevalence, disease burden and quality of life.

A RR protocol [17] was utilised to synthesise evidence in order to provide timely guidance to public policy and decision-making [18]. This approach has been adopted by several leading health organisations including the World Health Organisation [19] and the Canadian Agency for Drugs and Technologies in Health Rapid Response Service [20], to build a strong evidence base in a timely and accelerated manner, without compromising quality. A RR is not designed to be as comprehensive as a systematic review—it is purposive rather than exhaustive and provides actionable evidence to guide health policy [21].

**Fig. 1:**
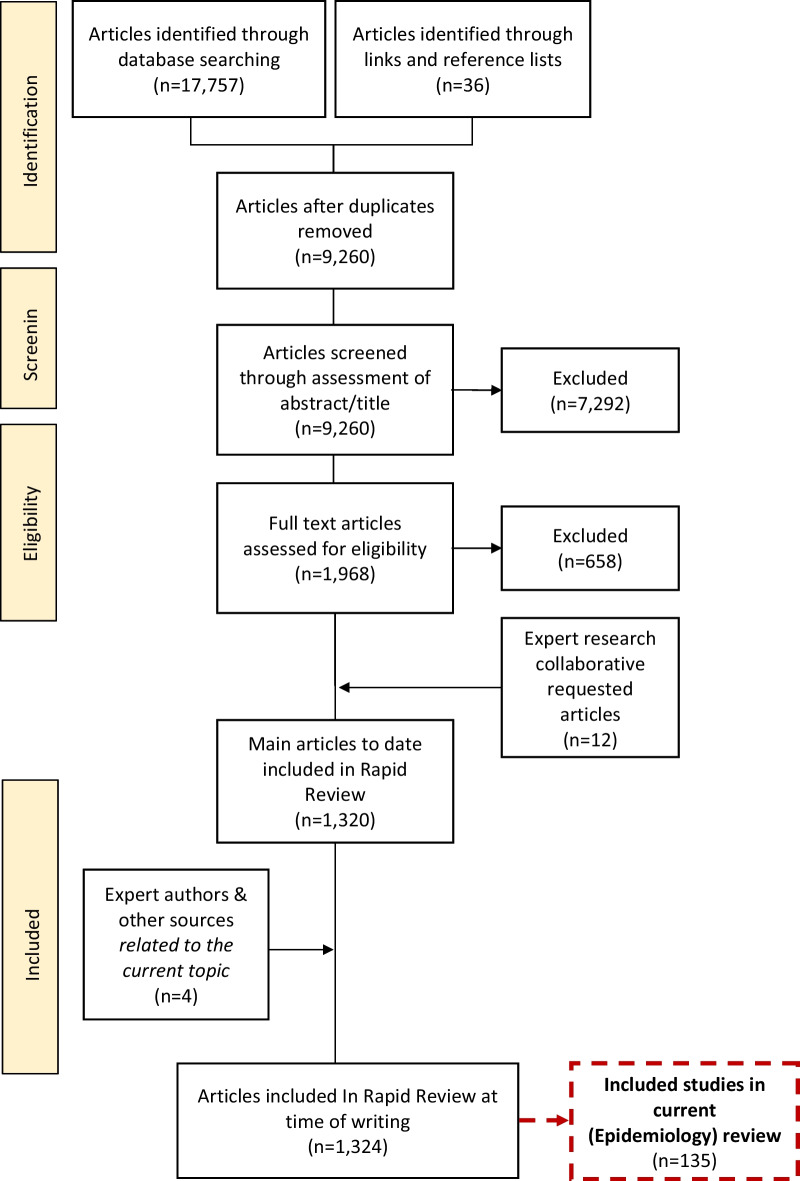
PRISMA flow-chart of included studies

The RR is a narrative synthesis and sought to adhere to the PRISMA guidelines [22]. It is divided by topic area and presented as a series of papers. Three research databases were searched: ScienceDirect, PubMed and Ovid/Medline. Included studies were published between 2009 and 2021, in English, and conducted within Western healthcare systems or health systems comparable to Australia in terms of structure and resourcing. Purposive sampling focused on high-level evidence studies such as: meta-analyses; systematic reviews; moderately sized randomised controlled studies (RCTs) (*n* > 50); moderately sized controlled-cohort studies (*n* > 50), and population studies (*n* > 500). Grey literature, such as clinical or practice guidelines, protocol papers (without results) and Masters’ theses or dissertations, was excluded. Instrument validation studies and studies commenting on the current *Diagnostic and Statistical Manual of Mental Disorders* (*DSM-5*) criteria for EDs were also excluded as they were not seen to be relevant to the patient-care focus of the review. Other sources included the personal libraries of authors, which yielded four additional studies (Fig. 1). This was conducted in line with the PRISMA-S: an extension to the PRISMA Statement for Reporting Literature Searches in Systematic Reviews [23].

Full methodological details including eligibility criteria, search strategy and terms, consort diagram, and data analysis are published in a separate protocol paper, which included a total of 1320 studies [10]. Data from included studies relating to Epidemiology are presented in the current review.

## Results

### Study characteristics and quality overview of included studies

#### Overview

The search identified 135 papers (Table 1) related to the epidemiology of EDs, of which four were meta-analyses of ED global prevalence, including in non-western populations [9, 24, 25, 26]. One systematic review was identified that provided prevalence ranges from reviewed studies [27]. The search also found 31 primary studies of prevalence which are summarised in Additional file 1: Table S1 (range of population measures for EDs) and Additional file 1: Table S2 (trial features). Findings comprised a wide range of studies conducted in both community-based samples as well as clinical samples. Thus, the estimates derived in this RR show a wide variance in reported prevalence and incidence rates. Other factors contributing to the varied ranges reported include different methods of measurement (e.g., self-report, diagnostic interviews or formal diagnoses obtained through health system registries) and study designs.

**Table 1:** Included studies

Author	Country	N	Population	Aim	Design	Outcome measure
Abebe et al. [28]	Norway	3844	Community (Mixed Cohort, All Genders)	To investigate age trends and gender difference in binge eating, purging and non-purging compensatory behaviours (CB) and the relationship of such behaviours to psychosocial problems	Longitudinal (< 5 years)	Binge eating, compensatory behaviours, purging
Ackard et al. [15]	USA	1040	Inpatient (Adult, Women)	To identify changes in the past 2 decades in the prevalence of middle-aged (MA; 40 + years) and young-adult (YA; 18–39 years) women seeking treatment for an eating disorder (ED) and to identify differences and similarities between both groups	Cross-sectional (Correlational)	Prevalence of eating disorder admissions by admission age from 1989 to 2006
Agh et al. [29]	Worldwide	69	Community (Mixed Cohort, All Genders)	To perform a systematic review of the health-related quality of life (HRQoL) and economic burdens of anorexia nervosa (AN), bulimia nervosa (BN), and binge eating disorder (BED)	Review (Systematic)	Health related quality of life (HRQoL) and economic burdens of AN, BN, and BED
Allen et al. [30]	Australia	1383	Community (Mixed Cohort, All Genders)	To compare the prevalence, stability, and psychosocial correlates of DSM–IV–TR and DSM-5 eating disorders, in a population-based sample of male and female adolescents followed prospectively from 14 to 20 years of age	Longitudinal (< 5 years)	Prevalence of DSM-5 AN, BED. BN, other eating disorder, OSFED (“other” eating disorders), EDNOS (Eating Disorder Not Otherwise Specified)
Austin et al. [31]	USA	13,795	Community (Adolescents, All Genders)	To describe patterns of purging and binge eating from early through late adolescence in female and male youth across a range of sexual orientations	Longitudinal (< 5 years )	Prevalence of purging and binge eating by sexual orientation
Baiano et al. [32]	Italy	80	Community (Adult, All Genders)	To analyze health-related quality of life (HRQoL) in subgroups of eating disorder (ED) patients by using the brief version of WHOQoL questionnaire (WHOQoL-BREF) before treatment administration	Cross-sectional (Correlational)	Health-related quality of life (HRQoL)
Baker and Runfola [33]	N/A	N/A	N/A	To provide an overview of the prevalence and challenges of midlife EDs and related symptomatology	Review (Narrative)	Prevalence of eating disorder diagnoses and binge-eating behaviours
Bould et al. [34]	Sweden	158,697	Community (Children, All Genders)	To investigate whether parental eating disorders (ED) predict ED in children, using a large multigeneration register-based sample	Cross-sectional (Correlational)	Parental eating disorder; eating disorder
Bourne et al. [35]	Worldwide	77	Community (Mixed Cohort, All Genders)	To systematically assess the extent and nature of the avoidant/restrictive food intake disorder (ARFID) literature	Review (Systematic)	Prevalence, clinical outcomes and characteristics of ED
Bryant-Waugh, [36]	N/A	1	Outpatient (Young People, Men)	To summarize current knowledge and clinical practice relating to ARFID in youth	Case Study	Knowledge and clinical practice relating to ARFID
Bueno et al. [37]	Spain	855	Inpatient (Adult, All Genders)	To compare the severity of eating disorders, eating disorder subtype, and personality profiles in a clinical sample of consecutively assessed women with eating disorders with later age of onset (LAO ≥ 25 years) to women with typical age of onset (TAO < 25 years)	Cross-sectional (Correlational)	Symptomology of eating disorders patients with late (late age onset ≥ 25 years) versus typical age of onset
Burke et al. [38]	USA	145,379	Community (Young Adult, All Genders)	To examine prevalence estimates of ED pathology across several distinct multiracial groups, to contrast prevalence estimates of ED pathology in each multiracial group with those among the corresponding monoracial identities, and to investigate these findings inter sectionally with gender identity	Cross-sectional (Correlational)	Prevalence estimates of ED pathology
Burt et al. [39]	Australia	6052	Community (Adult, All Genders)	To estimate the prevalence of eating disorders amongst First Australians at the diagnostic threshold level and to compare clinical features and health related quality of life (HRQoL) in First and other Australians with and without an eating disorder	Cross-sectional (Correlational)	Prevalence of ED; clinical features and health related quality of life
Calzo et al. [8]	Worldwide	52	N/A	To summarize trends and key findings from empirical studies conducted between 2011 and 2017 regarding eating disorders and disordered weight and shape control behaviours among lesbian, gay, bisexual, and other sexual minority (i.e., nonheterosexual) populations	Review (Systematic)	Sexual orientation disparities in eating disorder risk
Calzo et al. [40]	UK	5048	Community (Adolescents, All Genders)	To determine the associations of sexual orientation and eating disorder symptoms among adolescents in the UK	Cross-sectional (Correlational)	ED symptoms, body dissatisfaction
Carta et al. [41]	Italy	4999	Community (Adult, All Genders)	To evaluate the prevalence of eating disorders in an Italian community sample as well as to measure the burden of the quality of life of people and to compare it to those attributable to other chronic illnesses	Cross-sectional (Correlational)	Prevalence of ED; quality of life
Ng et al. [42]	England	2870	Community (Adult, All Genders)	To investigate: (a) the association of eating disorders with childhood sexual abuse and recent stressful life events; (b) the coexistence of eating disorders and other common psychiatric disorders; and (c) the impact of eating disorders on obesity, medical conditions, and health service utilization	Cross-sectional (Correlational)	12-Month prevalence of eating disorders; psychosocial risk factors; psychiatric disorder, physical health
Compte et al. [43]	Argentina	472	College (Adult, All Genders)	To assess the prevalence of eating disorder (ED) and muscle dysmorphia (MD) in male university students of Buenos Aires	Cross-sectional (Correlational)	Prevalence of eating disorders and muscle dysmorphia
Conceicao et al. [44]	Portugal	342	Community (Adult, Women)	To examine the point prevalence of eating disorders and picking/nibbling in elderly women	Cross-sectional (Correlational)	Prevalence of DSM-5 eating disorders and picking/nibbling in elderly women
Cooney et al. [45]	Canada	386	Outpatient (Young People, All Genders)	To determine the incidence of ARFID and describe the clinical and psychological characteristics of young people with ARFID undergoing eating disorder assessment in a tertiary care eating disorder program	Cross-sectional (Correlational)	Clinical and psychological features of avoidant/restrictive intake disorder
Cossrow et al. [46]	USA	22,397	Community (Adult, All Genders)	To estimate binge eating disorder (BED) prevalence according to DSM-5 and DSM-IV-TR criteria in US adults and to estimate the proportion of individuals meeting DSM-5 BED criteria who reported being diagnosed	Cross-sectional (Correlational)	3-Month, 12-month, and lifetime DSM-5 and DSM-IV-TR Binge eating disorder prevalence estimates
da Luz et al. [47]	Australia	1995: n = 3001, 2005 n = 3047, 2015: n = 3005	Community (Adult, All Genders)	To examine the prevalence of obesity and comorbid eating disorder behaviours from 2005 to 2015	Cross-sectional (Correlational)	Prevalence of obesity or binge eating, or obesity with comorbid binge eating; prevalence of very strict dieting/fasting; prevalence of purging, or obesity with comorbid purging
Dahlgren et al. [27]	Worldwide	19	Community (Mixed Cohort, All Genders)	To systematically review the literature on the prevalence of eating disorders (EDs) during the DSM-5 era, and to report rates of point- and lifetime prevalence	Review (Systematic)	Prevalence of ED
Darby et al. [48]	Australia	5366	Community (Adult, All Genders)	To measure the cooccurrence of obesity and eating disorder (ED) behaviours in the South Australian population and assess the change in level from 1995 to 2005	Cross-sectional (Correlational)	Population prevalence of comorbid obesity and ED behaviours
de Zwaan et al. [49]	Germany	2460	Community (Mixed Cohort, All Genders)	To estimate the prevalence and correlates of night eating syndrome (NES) in a large representative German sample using a validated self-report measure (NEQ) as the screening tool	Cross-sectional (Correlational)	Prevalence of night eating disorder
Dubovi et al. [50]	USA	144	College (Adult, All Genders)	To examine the extent to which the Big Five personality traits (openness, emotional stability, agreeableness, conscientiousness, and extraversion) would be associated with ED symptoms among college men	Cross-sectional (Correlational)	Personality traits and disordered eating symptoms
Duncan et al. [51]	USA	12,337	Community (Adult, All Genders)	To determine whether the prevalence of lifetime and past 12-month DSM-IV eating disorders (ED) diagnoses differed by body mass index category among men and women in a general population sample	Cross-sectional (Correlational)	Prevalence of lifetime and past 12-month DSM-IV eating disorders (ED) diagnoses
Eddy et al. [52]	USA	2231	Outpatient (Young People, All Genders)	To examine the prevalence of ARFID and inter-rater reliability of its diagnostic criteria in a paediatric gastrointestinal sample	Cross-sectional (Correlational)	Prevalence of ARFID
Elran-Barak et al. [53]	USA	2118	Community (Adult, All Genders)	To examine eating disorders in midlife and beyond by comparing frequency of anorexia nervosa (AN), bulimia nervosa (BN), binge eating disorder (BED), and other specified feeding or eating disorder (OSFED) among midlife eating disorder treatment-seeking individuals and younger controls, and to compare demographic and eating disorder-related characteristics across diagnoses and age groups	Cross-sectional (Correlational)	Frequency of anorexia nervosa (AN), bulimia nervosa (BN), binge eating disorder (BED), and other specified feeding or eating disorder (OSFED) among midlife eating disorder treatment-seeking individuals and younger control
Erskine et al. [54]	N/A	N/A	Community (Mixed Cohort, All Genders)	To present the GBD 2013 burden findings for anorexia nervosa and bulimia nervosa and explores the methodology underpinning these estimates	Modelling (Statistical)	YLLs, YLDs, and DALYs of ED
Erskine and Whiteford [26]	Worldwide	28	Community (Adult, All Genders)	To assess the available epidemiological data to determine whether BED should be considered for inclusion in global disease burden quantification efforts, such as the Global Burden of Disease Study (GBD)	Review (Narrative)	Global pooled prevalence of BED
Fairweather-Schmidt and Wade [55]	Australia	699	Community (Mixed Cohort, All Genders)	To determine the prevalence of specific DSM-5 eating disorders, and the proportion of Other Specified Feeding and Eating Disorders (OFSED) relative to threshold ED (TED) diagnoses, to examine whether key variables related to impairment distinguish people with TEDs from OSFED, compared with those without an eating disorder, and to investigate whether risk factors for the two eating disorder groups overlap with respect to both latent risk factors (additive genetic and environmental variance in relation to the threshold and OSFED phenotypes) and specific risk factors	Longitudinal (< 5 years)	Prevalence of DSM-5 eating disorders diagnosis
Feder et al. [56]	Canada	97	Outpatient (Young People, All Genders)	To describe ED presentations in youth presenting for gender dysphoria assessment and treatment	Cross-sectional (Correlational)	Prevalence and characteristic of eating disorders (EDs)and gender dysphoria (GD) in an adolescent population
Feldman and Meyer [57]	USA	388	Community (Adult, All Genders)	To examine the prevalence of psychiatric disorders among lesbian, gay, and bisexual (LGB) men with eating disorders	Cross-sectional (Correlational)	Prevalence of psychiatric disorders
Fischer et al. [58]	Switzerland	1514	Community (Adult, All Genders)	To increase knowledge about the clinical features of NES in a sample of 1514 young adults aged 18–26 years from the general population who participated in an anonymous Internet survey	Cross-sectional (Correlational)	Knowledge about night eating disorder
Fischer et al. [59]	USA	712	Outpatient (Young People, All Genders)	To evaluate the DSM-5 diagnosis of Avoidant/Restrictive Food Intake Disorder (ARFID) in children and adolescents with poor eating not associated with body image concerns	Cross-sectional (Correlational)	Avoidant/restrictive intake disorder; body image
Flament et al. [60]	Canada	3043	Community (Adolescents, All Genders)	To estimate jointly the point prevalence of weight and eating disorders in a community sample of adolescents; to investigate psychosocial correlates of thinness, overweight, and obesity, and of full- and subthreshold eating disorders (EDs); and to examine the relationships between weight status and prevalence of EDs	Cross-sectional (Correlational)	Point prevalence of DSM-5 AN, BN, BED, PD, and subthreshold AN and BN; weight status; subthreshold AN and BN
Folope et al. [61]	France	130	Community (Adult, All Genders)	To identify specific factors involved in the poor quality of life (QoL) of obese subjects, such as psychological distress (anxiety and depression disorders), eating disorders (EDs), impaired body image perception, and physical health difficulties	Cross-sectional (Correlational)	Quality of life (QoL); psychological distress; eating disorders (EDs); impaired body image perception; physical health difficulties
Fornaro et al. [62]	Worldwide	47	Community (Mixed Cohort, All Genders)	To assess the prevalence and clinical correlates of BD ⇌ ED across the lifespan	Cross-sectional (Correlational)	Prevalence of BD and ED
Galmiche et al. [9]	Worldwide	94	Community (Mixed Cohort, All Genders)	To report the prevalence of the different EDs or total EDs and to study their evolution	Review (Systematic)	Prevalence of all EDs
Gammelmark et al. [63]	Denmark	N/A	Community (Mixed Cohort, All Genders)	To investigate if the increase in incidence of eating disorders previously shown in Denmark and internationally in secondary healthcare has continued in Denmark until recent years	Cross-sectional (Correlational)	Incidence of ED
Gatt et al. [64]	Australia	90	Community (Mixed Cohort, All Genders)	To investigate the household economic burden of eating disorders and cost-related non-adherence to treatment in Australia	Cross-sectional (Correlational)	Household economic burden of eating disorders; adherence to treatment
Gauvin et al. [65]	Canada	1501	Community (Adult, Women)	To estimate the prevalence of eating disorders and maladaptive eating behaviours in a population-based sample and examined the association of maladaptive eating with self-rated physical and mental health	Cross-sectional (Correlational)	Observed proportions, observed frequencies [95% confidence intervals (CI)], weighted prevalence estimates (95% CIs), and estimated number of women affected by various eating disorder symptoms and syndromes
Kyu et al. [66]	Worldwide	N/A	Community (Mixed Cohort, All Genders)	To present global burden disease (GBD) 2017 results for healthy life expectancy (HALE) and disability-adjusted life-years (DALYs) by age and sex from 1990 to 2017 for 195 countries and territories	Modelling (Statistical)	Prevalence of ED
Goldberg et al. [67]	Canada	190	Inpatient (Young Adult, Women)	To determine the prevalence of child and adolescent females at risk for Avoidant Restrictive Food Intake Disorder (ARFID) in a tertiary care paediatric and adolescent gynaecology (PAG) clinic	Cross-sectional (Correlational)	Prevalence of ARFID
Griffiths et al. [68]	Australia and New Zealand	2733	Community (Adult, Men)	To examine the associations of anabolic androgenic steroids (AAS) use, and of thoughts about using AAS, with body image, eating disorder symptoms, and quality of life among gay and bisexual men living in Australia and New Zealand	Cross-sectional (Correlational)	Anabolic steroid use
Hammerle et al. [69]	Germany	1654	Community (Adolescents, All Genders)	To investigate, for the first time in Germany, adolescent prevalence rates of DSM-5 eating disorders	Cross-sectional (Correlational)	Prevalence of full syndrome adolescent anorexia nervosa, bulimia nervosa, binge eating disorder and other specified feeding or eating disorder (OSFED), partial and subthreshold eating disorders
Harrop et al. [70]	Worldwide	75	Community (Mixed Cohort, All Genders)	To assess atypical anorexia nervosa (AAN) literature from 2007 to 2020, to investigate: (a) the demographic characteristics of AAN studies, (b) the prevalence of AAN compared with AN, (c) the range of operational definitions of AAN and the implications of these definitions, and (d) the proportion of patients with AAN and AN represented in consecutive admission and referral samples	Systematic Review/ Meta-Analysis (combined)	Prevalence of AN and AAN
Hay and Carriage [71]	Australia	2005: n = 3047, 2008: n = 3034	Community (Adult, All Genders)	To investigate the current 3-month prevalence of eating disorder behaviours of binge eating, restrictive dieting, and extreme weight control methods such as vomiting, and core eating disorder psychopathology of excessive weight and shape concerns, in a representative general population sample of older adolescent and adult indigenous Australians	Cross-sectional (Correlational)	Prevalence of eating disorder features
Hay et al. [72]	Australia	2014: n = 2732, 2015: n = 3005	Community (Mixed Cohort, All Genders)	To extend previous research on the prevalence, burden and HRQoL of people with eating disorders in the South Australian population in new samples who were surveyed in 2014 and 2015	Cross-sectional (Correlational)	3-Month prevalence of anorexia nervosa-broad, bulimia nervosa and ARFID
Hay et al. [73]	Australia	6041	Community (Adult, All Genders)	To explore the demographic correlates of these disorders, specifically, age, gender, income, and educational attainment and presence of obesity	Cross-sectional (Correlational)	Point (3-month) prevalence of anorexia nervosa and bulimia nervosa
Heriseanu et al. [74]	Australia	3047	Community (Adult, All Genders)	To report on the distribution of compulsive grazing (CG) and non-compulsive grazing (NCG) in the Australian population, and to assess associations with obesity, ED, and health-related functioning	Cross-sectional (Correlational)	Distribution of compulsive grazing (CG) and non-compulsive grazing (NCG)
Hughes et al. [75]	Australia	3270	Community (Adolescents, All Genders)	To estimate the population prevalence of eating disorder symptoms in relation to weight status in adolescents	Cross-sectional (Correlational)	Estimated population prevalence of AN and BN symptoms
Isomaa et al. [76]	Finland	595	Community (Adolescents, All Genders)	To investigate the prevalence, incidence and development of eating disorders and subclinical eating pathology	Repeated Measure (with follow-up)	Prevalence and eating pathology of DSM-IV eating disorders diagnosis
Jaite et al. [77]	Germany	1404	Outpatient (Young People, All Genders)	To investigate the prevalence, psychiatric comorbidity and outpatient treatment in a sample of German children and adolescents with eating disorders (ED)	Cross-sectional (Correlational)	Prevalence of AN and BN
Javaras et al. [78]	Sweden	2.3 million	Community (Mixed Cohort, All Genders)	To investigate the sex- and age-specific incidence of healthcare register-recorded anorexia nervosa (AN) and other eating disorders (OED) in a complete birth cohort, and assess whether incidence varies by diagnostic period and (sub-) birth cohort	Cross-sectional (Correlational)	Incidence of AN and OED
Jenkins et al. [79]	N/A	N/A	Community (Adult, All Genders)	To provide a summary of research into eating disorder-related quality of life (EDQoL), with a focus on what variables might affect the relationship between ED pathology and QoL	Review (Narrative)	Eating disorder-related quality of life (EDQoL); ED pathology
Kambanis et al. [80]	USA	343	Community (Adult, All Genders)	To clarify the utility of (Amazon’s Mechanical Turk) MTurk as an ED data collection alternative	Cross-sectional (Correlational)	ED psychopathology
Keski-Rahkonen et al. [81]	Finland	2881	Community (Adult, Women)	To report the incidence, prevalence and outcomes of bulimia nervosa using for the first time a nationwide study design	Cross-sectional (Correlational)	Incidence, prevalence and outcomes of bulimia nervosa
Kessler et al. [24]	Worldwide	24,124	Community (Adult, All Genders)	To present cross-national BED data and compare with bulimia nervosa (BN) data in the World Health Organization (WHO) World Mental Health Surveys	Cross-sectional (Correlational)	Prevalence of BN and BED; age of onset and persistence; comorbidity with other mental health; role impairment
Kovacic et al. [82]	USA	N/A	Community (Children, All Genders)	To examine the population-wide prevalence of paediatric feeding disorder (PFD) in the US, track this prevalence over several years, and compare the prevalence of PFD among the different databases in children greater than 2 months of age	Longitudinal (> 10 years)	Prevalence of Paediatric Feeding Disorder
Krom et al. [83]	Netherlands	48	Outpatient (Young People, All Genders)	To compare health related quality of life (HRQOL) in infants and children with avoidant restrictive food intake disorder (ARFID) to healthy and chronically ill controls	Cross-sectional (Correlational)	Health related quality of life (HRQOL)
Kurz et al. [84]	Switzerland	1444	Community (Children, All Genders)	To determine the distribution of early-onset restrictive eating disturbances characteristic of the new DSM-5 diagnosis, avoidant/restrictive food intake disorder (ARFID) in middle childhood, as well as to evaluate the screening instrument, Eating Disturbances in Youth-Questionnaire (EDY-Q)	Cross-sectional (Correlational)	Early-onset restrictive eating disturbances characteristics of AFID
Lähteenmäki et al. [85]	Finland	1863	Community (Young Adult, All Genders)	To investigate the epidemiology of eating disorders in a population-based sample of young adults	Cross-sectional (Correlational)	Lifetime prevalence of anorexia nervosa, bulimia nervosa, eating disorder not otherwise specified and any eating disorder; comorbidity; treatment contacts and medication;
Lapid et al. [86]	Worldwide	48	N/A	To review all existing literature on eating disorders in the elderly and provide practical guidelines for clinicians in recognizing and managing eating disorders in the elderly	Review (Narrative)	Cases of eating disorders in people over the age of 50 years; comorbid psychiatry condition; intervention received
Larranaga et al. [87]	Spain	N/A	Community (Mixed Cohort, All Genders)	To determine the incidence and prevalence of eating disorder and its clinical forms	Cross-sectional (Correlational)	Incidence and prevalence of eating disorder
Le et al. [88]	Australia	N/A	Community (Adolescents, Girls)	To evaluate the modelled population cost-effectiveness of cognitive dissonance (CD), a school-based preventive intervention for EDs, in the Australian health care context	Modelling (Statistical)	Cost-effectiveness of cognitive dissonance
Le Grange et al. [89]	USA	13,103	Community (Mixed Cohort, All Genders)	To examine prevalence and clinical correlates of eating disorder not otherwise specified (EDNOS) in the US population	Cross-sectional (Correlational)	Prevalence and clinical severity of DSM-IV EDNOS diagnosis
Madden et al. [90]	Australia	101	Inpatient (Young People, All Genders)	To collect nationally representative epidemiological data on early onset eating disorders (EOEDs) in children	Cross-sectional (Correlational)	Early-onset eating disorders (EOEDs) rates, clinical features and complications, hospitalisation, psychological comorbidity, and concordance of clinical features with DSM-IV criteria
Mancuso et al. [91]	Australia	117	Outpatient (Adult, All Genders)	To examine the relative prevalence rates of DSM-IV and DSM-5 eating disorder diagnoses in a large sample of patients with a broad range of diagnoses who presented for treatment in a community out-patient setting	Cross-sectional (Correlational)	Prevalence of DSM-IV and DSM-5 eating disorder diagnoses
Mangweth-Matzek et al. [92]	Austria	715	Community (Adult, Women)	To assess eating behaviour and body image in 715 community women aged 40–60 in Innsbruck, Austria	Cross-sectional (Correlational)	Prevalence and body images of women with eating disorders, subthreshold eating disorders, and normal eating
Mangweth-Matzek et al. [92]	Austria	436	Community (Adult, Women)	To explore the association between menopausal status and eating disorders, including associated pathology, in a large population of women between 40 and 60 years of age	Cross-sectional (Correlational)	Prevalence of eating disorder; body Weight; weight control; eating behaviour; body Image in middle-aged women
Martin et al. [93]	Spain	528	Outpatient (Adult, All Genders)	To analyse the quality of life (QoL) of a broad sample of patients with eating disorders (ED) and to identify potential factors that predict QoL	Cross-sectional (Correlational)	Quality of life
Martínez-González et al. [94]	Worldwide	31	Community (Mixed Cohort, All Genders)	To summarize the incidence of AN using a systematic review and meta-analysis	Systematic Review/ Meta-Analysis (combined)	Incidence of AN
Masheb and White [95]	USA	1897	Community (Adult, Women)	To examine overweight bulimia nervosa (BN) in a community sample of women	Cross-sectional (Correlational)	Rate of overweight BN
Meneguzzo et al. [96]	Worldwide	372,256	Community (Mixed Cohort, Girls)	To investigate the available literature regarding lesbian and bisexual women, in order to better understand the occurrence of eating disorder psychopathology in sexual minorities women	Review (Systematic)	Eating disorders symptoms
Micali et al. [97]	UK	5658	Community (Adult, Women)	To investigate the lifetime and 12-month prevalence of EDs and lifetime health service use and to identify childhood, parenting, and personality risk factors	Cross-sectional (Correlational)	Lifetime and 12-month prevalence of eating disorders; risk factors
Midlarsky et al. [98]	USA	245	Community (Adult, Women)	To systematically explore the association between psychological factors found to be correlated with eating disorders in both younger and middle-aged women and eating pathology in later life	Cross-sectional (Correlational)	Disordered eating symptomatology; body dissatisfaction; aging-related concern about appearance; sociocultural to be thin; perfectionism; life stress; depression
Mitchison et al. [99]	Australia	1998: n = 3010, 2008: n = 3034	Community (Mixed Cohort, All Genders)	To examine temporal differences to the demographic correlates of eating disorder behaviours over a 10-year period	Cross-sectional (Correlational)	Prevalence of objective binge eating, extreme dieting, and purging; quality of life
Mitchison et al. [100]	Australia	828	Community (Adult, Women)	To provide a first-time investigation of possible bidirectional relationships between EDs and both health related QoL (HRQoL) and psychological distress (PD)	Longitudinal (> 5 years)	Health-related quality of life; psychological distress
Mitchison et al. [101]	Australia	15,126	Community (Adult, All Genders)	To assess the time trends in binge-eating prevalence and burden over 18 years	Cross-sectional (Correlational)	Point (3-month) prevalence of objective binge eating (OBE), health-related quality of life, days out of role, and distress related to OBE
Mitchison et al. [102]	Australia	5191	Community (Adolescents, All Genders)	To provide the first prevalence report of the full suite of DSM-5 eating disorders in adolescence, and to examine the impact of applying a criterion for clinical significance	Cross-sectional (Correlational)	Prevalence rates from 1998 to 2008 in binge eating, extreme dieting, and purging; mental health-related quality of life;
Mohler-Kuo et al. [103]	Switzerland	10,038	Community (Mixed Cohort, All Genders)	To generate updated lifetime and 12-month prevalence estimates for EDs, taking into consideration the three main diagnoses (AN, BN, BED) and two sub-threshold categories (sub-threshold BED and any binge eating), using a large national representative sample in Switzerland	Cross-sectional (Correlational)	Prevalence of AN, BN, BED, sub-threshold ED, any AN, any Binge eating; age of onset; professional help-seeking; mental health outcomes (SMI, social phobia, and quality of life)
Mond et al. [104]	Australia	159	Community (Adult, Women)	To examine impairment in quality of life in a community sample of women with eating disorders recruited as part of an epidemiological study	Cross-sectional (Correlational)	Quality of life
Murray et al. [7]	N/A	N/A	N/A	To provide a synthesis of relevant research relating to a multitude of dimensions of disordered eating in males	Review (Narrative)	History and characteristics of male presentations of eating disorders (EDs)
Mustelin et al. [105]	Finland	2825	Community (Mixed Cohort, Girls)	To assess the population prevalence and incidence of binge eating disorder (BED) among young women	Longitudinal (> 10 years)	Prevalence and incidence of binge eating disorder (BED)
Mustelin et al. [106]	Finland	5248	Community (Young Adult, All Genders)	To investigate the prevalence of features of BED and their association with body mass index (BMI) and psychological distress among men and women from the longitudinal community based FinnTwin16 cohort (born 1975–1979)	Longitudinal (< 5 years)	Lifetime prevalence estimates and incidence rates of DSM-5 OSFED and UFED; comorbidity; course of illness
Mustelin et al. [106]	Finland	2825	Community (Adult, Women)	To examine the occurrence, course, and clinical picture of the DSM-5 residual categories ‘Other Specified Feeding or Eating Disorder’ (OSFED) and ‘Unspecified Feeding or Eating Disorder’ (UFED), to describe potential subtypes, and to evaluate whether the subdivision of the residual category appears meaningful	Cross-sectional (Correlational)	Lifetime prevalence of DSM-5 OSFED/UFED diagnosis
Nagata et al. [107]	N/A	N/A	Community (Mixed Cohort, All Genders)	To review the recent literature on eating disorders, disordered eating behaviors (DEB), and body image dissatisfaction among sexual and gender minority populations, including, but not limited to, gay, lesbian, bisexual, and transgender people	Review (Narrative)	ED in sexual minority individuals
Nagl et al. [108]	Germany	3021	Community (Adolescents, All Genders)	To assess the prevalence, incidence, age-of-onset and diagnostic stability of threshold and subthreshold anorexia nervosa (AN) and bulimia nervosa (BN) in the community	Longitudinal (< 5 years)	Lifetime prevalence at baseline and cumulative lifetime incidences of ED
Nicely et al. [109]	USA	173	Outpatient (Young People, All Genders)	To determine the prevalence of ARFID in children and adolescents undergoing day treatment for an eating disorder, and to compare ARFID patients to other eating disorder patients in the same cohort	Cross-sectional (Correlational)	Prevalence of Avoidant/restrictive intake disorder
Nicholls et al. [110]	UK and Ireland	505	Outpatient (Young People, All Genders)	To identify new cases of early-onset eating disorders (< 13 years) presenting to secondary care over 1 year and to describe clinical features, management and 1-year outcomes	Longitudinal (< 5 years)	Clinical features and management of early onset eating disorders
Olsen et al. [111]	Denmark	2509	Community (Adolescents, All Genders)	To estimate the prevalence of BED in community adolescents and explore the significance of BED at this age by investigating the correlations with concurrent overweight, mental health problems, self-reported impact, and socio-economic risk factors	Cross-sectional (Correlational)	Prevalence of BED; overweight, mental health problems, self-reported impact, and socio-economic risk factors
Pasold and Portilla [112]	USA	507	Inpatient (Young People, Women)	To explore trends among patients presenting to an outpatient multidisciplinary child and adolescent eating disorders program over a period of 14 years (1997–2010)	Cross-sectional (Correlational)	Prevalence of AN, BN and EDNOS
Perez and Warren [113]	USA	20,013	Community (Adult, All Genders)	To examine the relationship between obesity status, binge-eating disorder (BED), and quality of life (QOL) in a large, ethnically diverse community sample of adult men and women	Cross-sectional (Correlational)	Obesity status; binge-eating disorder (BED); quality of life (QOL)
Pinhas et al. [114]	Canada	2453	Outpatient (Young People, All Genders)	To document and describe the incidence and age-specific presentation of early-onset restrictive eating disorders in children across Canada	Cross-sectional (Correlational)	Incidence and age-specific presentation of early-onset restrictive eating disorders
Preti et al. [115]	Europe	21,425	Community (Adult, All Genders)	To investigate the prevalence of non-psychotic mental disorders in six European countries (Belgium, France, Germany, Italy, the Netherlands and Spain), using a new version of the Composite International Diagnostic Interview	Cross-sectional (Correlational)	Lifetime estimated prevalence of anorexia nervosa, bulimia nervosa, binge eating disorder, sub-threshold binge eating disorder, and any binge eating
Qian et al. [25]	Worldwide	33	Community (Mixed Cohort, All Genders)	To update the prevalence of eating disorders in the general population before 2021 and to analyse the distribution characteristics at different times and in different regions and sexes, as well as the diagnostic criteria	Systematic Review/ Meta-Analysis (combined)	Prevalence of ED
Reas and Ro [116]	Norway	N/A	Community (Mixed Cohort, All Genders)	To investigate time trends in the age- and sex-specific incidence of healthcare detected anorexia nervosa (AN) and bulimia nervosa (BN) from 2010 to 2016	Cross-sectional (Correlational)	Incidence of AN and BN
Ribeiro et al. [117]	Portugal	805	College (Adult, All Genders)	To assess the prevalence of BED among college students using a two-stage design	Cross-sectional (Correlational)	Prevalence of binge eating disorder
Rozzell et al. [118]	USA	4524	Community (Children, All Genders)	To report the prevalence rates of anorexia nervosa (AN), bulimia nervosa (BN), binge eating disorder (BED), and other specified feeding and eating disorders (OSFED) in addition to a global “any ED” diagnosis, using Diagnostic and Statistical Manual of Mental Disorders (Fifth Edition) (DSM-5) criteria among a US representative sample of children aged 9 and 10 years	Cross-sectional (Correlational)	Prevalence rates of anorexia nervosa (AN), bulimia nervosa (BN), binge eating disorder (BED), other specified feeding and eating disorders (OSFED), and a global “any ED” diagnosis
Runfola et al. [119]	USA	1636	Community (Mixed Cohort, All Genders)	To compare students with and without NES on eating disorder symptomatology, quality of life, and mental health, while exploring the role of binge eating in associations	Cross-sectional (Correlational)	Eating disorder symptomatology; quality of life; mental health
Santomauro et al. [120]	Worldwide	54	Community (Mixed Cohort, All Genders)	To estimate the prevalence and burden of binge-eating disorder and other specified feeding or eating disorder (OSFED) globally and present a case for their inclusion in global burden of disease (GBD)	Modelling (Statistical)	Prevalence and burden (YLLs, YLDs, DALYs) of binge-eating disorder and OSFED
Shu et al. [121]	Australia	704	Inpatient (Young People, Men)	To provide knowledge about the clinical presentation of eating disorders in young males	Cross-sectional (Correlational)	Prevalence and clinical characteristics of ED in young males
Silen et al. [122]	Finland	1347	Community (Young Adult, Women)	To assess the detection, treatment and outcomes of DSM‐5 eating disorders in a nationwide community setting	Cross-sectional (Correlational)	Detection, treatment and outcomes of DSM‐5 eating disorders
Smink et al. [123]	Netherlands	2230	Community (Mixed Cohort, All Genders)	To establish the prevalence and severity of eating disorders based on the new DSM-5 criteria in a community cohort of adolescents	Longitudinal (< 5 years)	Prevalence of DSM-5 eating disorders
Sparti et al. [124]	Australia	2298	Community (Adolescents, All Genders)	To estimate the prevalence of disordered eating (DE) among Australian adolescents and examine associations with clinical mental health problems, problems with functioning, and help received	Cross-sectional (Correlational)	Prevalence of Disordered eating and lifetime eating disorder; Clinical mental health problem; problem with functioning; help seeking
Stoving et al. [125]	Denmark	605	Outpatient (Young People, All Genders)	To compare remission rates in purging and non-purging females with anorexia nervosa (AN) and eating disorder not otherwise specified (EDNOS) in a large retrospective single centre cohort	Cross-sectional (Correlational)	Remission, Purging behaviour
Streatfeild et al. [126]	USA	N/A	N/A	To estimate 1-year costs of eating disorders in the United States (U.S.) from a societal perspective, including the costs to the U.S. health system, individual and family productivity costs, lost wellbeing, and other societal economic costs, by setting and payer	Review (Other)	One-year cost of ED
Striegel-Moore et al. [127]	USA	5522	Community (Adult, All Genders)	To examine gender differences in prevalence of eating disorder symptoms including body image concerns (body checking or avoidance), binge eating, and inappropriate compensatory behaviours	Cross-sectional (Correlational)	Prevalence of eating disorder symptoms in women and men
Striegel Weissman and Rosselli [128]	N/A	N/A	N/A	To offer an update of the research literature published since 2011 in three research areas that undergirded the researchers' collaborative research project: unmet treatment needs, cost of illness, and cost-effectiveness of treatments	Review (Narrative)	Prevalence of ED
Swanson et al. [129]	USA	10,123	Community (Adolescents, All Genders)	To examine the prevalence and correlates of eating disorders in a large, representative sample of US adolescents	Cross-sectional (Correlational)	Lifetime prevalence estimates of AN, BN, and BED; age of onset; comorbidity with other mental disorder; social impairment; lifetime suicidality
Tannous et al. [130]	Australia	2977	Community (Mixed Cohort, All Genders)	To understand economic and other costs of EDs at the community level	Cross-sectional (Correlational)	Economic cost of BN and BED
Tholin et al. [131]	Sweden	21,741	Community (Adult, All Genders)	To assess the prevalence of night eating (NE) and associated symptoms in a population-based sample of Swedish twins	Cross-sectional (Correlational)	Prevalence of night eating disorder
Treasure et al. [132]	N/A	N/A	N/A	To describe the epidemiology, diagnosis, screening and prevention, aetiology, treatment and quality of life of patients with AN	Review (Narrative)	Prevalence of AN
Udo and Grilo [133]	USA	36,306	Community (Adult, All Genders)	To provide lifetime and 12-month prevalence estimates of DSM-5–defined anorexia nervosa (AN), bulimia nervosa (BN), and binge-eating disorder (BED) from the 2012–2013 National Epidemiologic Survey on Alcohol and Related Conditions	Cross-sectional (Correlational)	Prevalence estimates of lifetime AN, BN, and BED
Vallance et al. [134]	New Zealand	214	Community (Adult, All Genders)	To examine the impact of eating disorder psychopathology on quality of life (QOL) within a community sample	Cross-sectional (Correlational)	Eating disorder psychopathology; quality of life
van Hoeken and Hoek [135]	Worldwide	N/A	Community (Mixed Cohort, All Genders)	To review the recent literature on the burden of eating disorders in terms of mortality, disability, quality of life, economic cost, and family burden, compared with people without an eating disorder	Review (Narrative)	Mortality, disability, costs, quality of life, and family burden
Ward et al. [136]	USA	100,000	Community (Adult, All Genders)	To model the individual-level disease dynamics of ED from birth to age 40 years and to estimate the association of increased treatment coverage with ED-related mortality	Modelling (Statistical)	Age-specific 12 month and lifetime prevalence of ED; number of deaths per 100,000 general populations by age 40
Watson et al. [137]	Norway	77,267	Community (Adult, Women)	To internally validate previously published rates of remission, continuation and incidence of broadly defined eating disorders during pregnancy in the Norwegian Mother and Child Cohort (MoBa) at the Norwegian Institute of Public Health	Cross-sectional (Correlational)	Remission, continuation and incidence of eating disorders during early pregnancy
Watson et al. [138]	USA	26,002	Community (Adolescents, All Genders)	To document trends in disordered eating behaviours over the span of 14 years using a population-based sample to identify trends in disordered eating for heterosexual, bisexual, gay, and lesbian youth separately for males and females in Massachusetts	Longitudinal (> 10 years)	Prevalence and trends of 30-days use of diet pills and purging to lose weight by sexual orientation and sex
Watson et al. [139]	USA	55,597	Community (Adolescents, All Genders)	To explore the trends in unhealthy weight control behaviour (UWCB) among sexually active sexual minority youth identified using a measure of sexual behaviour	Longitudinal (> 10 years)	Unhealthy weight control behaviours
Weigel et al. [140]	Germany	218	Community (Mixed Cohort, Girls)	To examine the association between disorder specific factors, comorbidity and health related quality of life (HRQoL) in anorexia nervosa (AN)	Cross-sectional (Correlational)	Comorbidity and health related quality of life (HRQoL) in AN
Winkler et al. [141]	Worldwide	7	Community (Adult, All Genders)	To explore the differences in health-related quality of life (HRQoL) between AN, BN, EDNOS and BED, measured by the Medical Outcomes Study Short Form-36 Health Survey (SF-36)	Systematic Review/ Meta-Analysis (combined)	Health-related quality of life (HRQoL) between AN, BN, EDNOS and BED
Wong and Hay [142]	Australia	6041	Community (Mixed Cohort, All Genders)	To investigate the age of onset of EDBs and their enduring impact on mental health related quality of life (MHRQoL) and role impairment in a representative community sample	Cross-sectional (Correlational)	Age of onset of EDBs; mental health related quality of life
Wu et al. [143]	N/A	N/A	Community (Mixed Cohort, All Genders)	To reveal the burden of eating disorders at the global, regional and national levels using the Global Burden of Disease (GBD) Study 2017 data	Modelling (Statistical)	Prevalence and disability-adjusted life years of ED
Zeiler et al. [144]	Austria	3610	Community (Adolescents, All Genders)	To investigate the prevalence of eating disorder (ED) risk as well as associated psychopathology and health-related quality of life (HrQoL) in a large population sample of Austrian adolescents	Cross-sectional (Correlational)	Health-related quality of life (HRQoL)
Zerwas et al. [145]	Denmark	966,141	Inpatient and Outpatient (Adult, All Genders)	To characterize the incidence rates and cumulative incidence of anorexia nervosa (AN), bulimia nervosa (BN), and eating disorder not otherwise specified (EDNOS), and examine associations among eating disorder diagnoses, suicide attempts, and mortality	Cross-sectional (Correlational)	Incidence rates and cumulative incidence of AN, BN, and EDNOS; suicide attempts; mortality
Zerwas et al. [146]	Denmark	930,977	Community (Mixed Cohort, All Genders)	To investigate associations between autoimmune and autoinflammatory diseases and eating disorders in youth in a nationwide, population-based cohort	Cross-sectional (Correlational)	Autoimmune and autoinflammatory diseases; ED diagnosis
Zickgraf et al. [147]	USA	22	Outpatient (Young People, All Genders)	To describe the clinical characteristics of children, adolescents, and young adults diagnosed with the selective/neophobic presentation of ARFID in a non-eating disorder-focused outpatient setting, including demographics (age, gender), psychological and medical comorbidities, age of onset, symptom trajectory (history of adding or eliminating foods), and qualitative descriptions of psychosocial interference described by patients and families, and to explore the prevalence of each of the four components of Criterion A for ARFID (weight loss/difficulty gaining weight, nutritional deficiency, supplement use, and psychosocial interference) using strict and more expansive definitions of the three weight/nutrition criteria	Cross-sectional (Correlational)	Clinical characteristics of avoidant restrictive intake disorder
Zulig et al. [148]	USA	2242	Community (Adolescents, All Genders)	To investigate the relationship between selected disordered eating behaviours and self-reported sexual minority status (gay/lesbian, bisexual, and unsure) among a representative sample of high school adolescents	Cross-sectional (Correlational)	Eating behaviours by sexual orientation

**Fig. 2:**
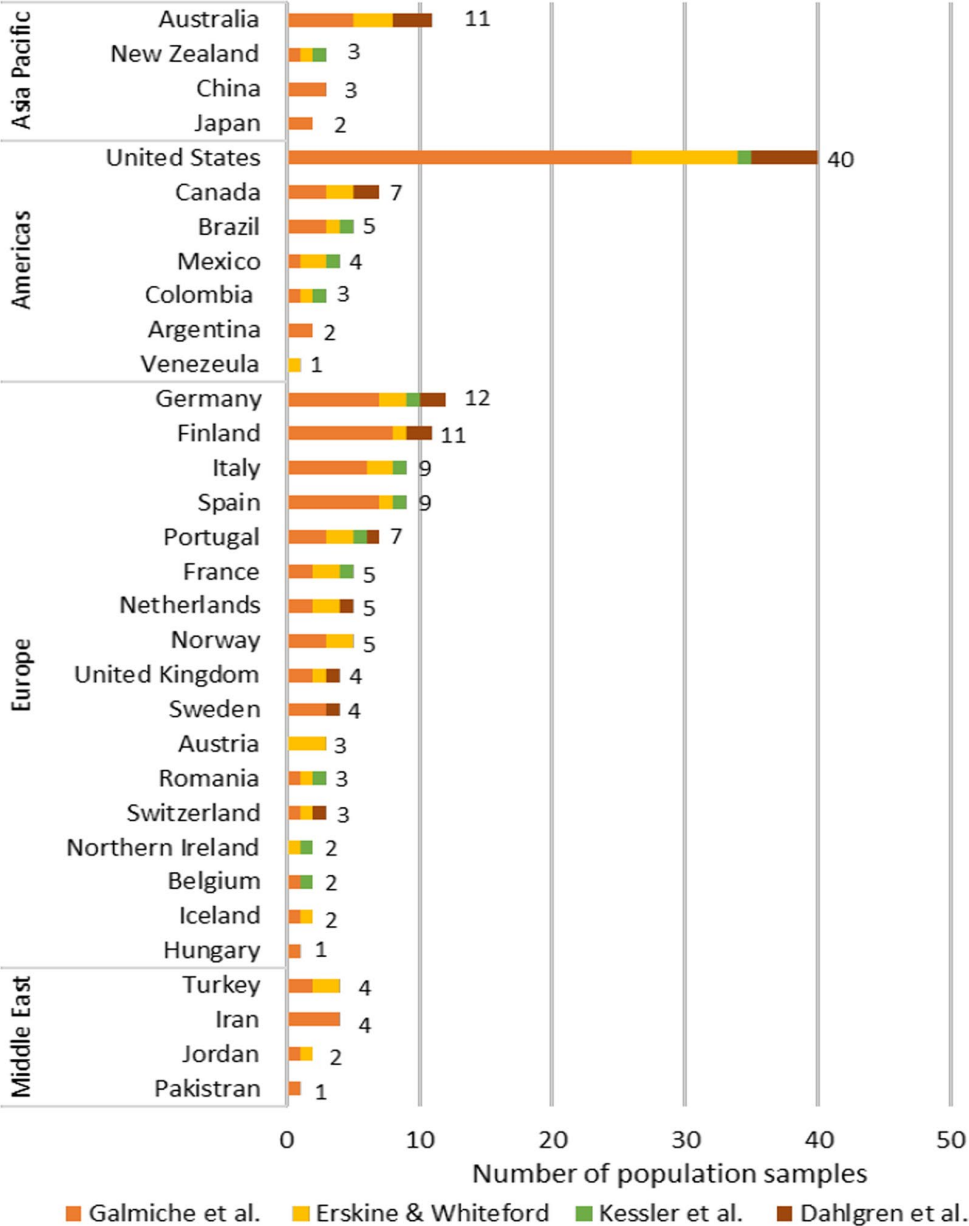
Countries included in meta-analyses and review of global prevalence rates

Sampled populations were from predominately developed Western countries, with a majority of studies (N = 179) coming from the United States of America (n = 40, 22.3%), Europe (n = 87, 48.6%), and Australia (n = 11, 6.1%). Figure 2 presents a breakdown of included studies by country.

#### Diversity of study design and quality considerations

Additional file 1: Table S1 highlights that, as may be expected, studies that used self-report to identify cases reported higher prevalence and those that employed interviews demonstrate more consistent prevalence rates. Of the included reviews, Dahlgren et al. [27] presented prevalence across design types examining studies that used a 2-stage design, interview and self-report data. Galmiche et al. [9] commented that although the majority (51%) of included studies in their meta-analyses (shown in Table 2) did not present pooled data by study design, the majority had used an interview assessment. Notably, 13% used the Structured Clinical Interview for DSM Disorders (SCID); 12% the Composite International Diagnostic Interview (CIDI); and 11% the Eating Disorder Examination. Furthermore, changes to diagnostic criteria have contributed to shifting prevalence rates even within the same study population.

**Table 2:** ED global prevalence from four meta-analyses and a systematic review

Study	Years	Prev	AN (%)	BN (%)	BED (%)	EDNOS/OSFED, UFED (%)	Any ED (%)
Erskine and Whiteford [26, 54]	2018	Point	–	–	1.1 F: 1.5 M: 0.4	–	–
		12-Month	–	–	0.7 F: 1.4 M: 0.6	–	–
Kessler et al. [24]	2013	Lifetime	–	1.0	1.9	–	–
		12-Month	–	0.4	0.8	–	–
Galmiche et al. [9]	2019	Lifetime	F: 1.4 M: 0.2	F: 1.9 M: 0.6	F: 2.8 M: 1.0	F: 4.3 M: 3.6	F: 8.4 M: 2.2
		Point	F: 2.8 M: 0.3	F: 1.5 M: 0.1	F: 2.3 M: 0.3	F: 10.1 M: 0.9	F: 5.7 M: 2.2
Qian et al. [25]	2021	Lifetime	0.16 F: 0.03 M: 0.01	0.63 F:1.22 M: 0.38	1.53 F: 1.17 M: 0.80		0.91 F:2.58 M:0.74
		12-Month	0.02 F: 0.62 M: 0.04	0.31 F: 0.29 M: 0.09	0.93 F: 0.93 M: 0.51		0.43 F: 0.93 M: 0.22
Dahlgren et al. [27]*	2017	Lifetime	0.1–1.4	2.6	2.7–3.6	OSFED: 3.8–11.5 UFED: No Data	No data
		Point	0.5–2.0	0.7–1.0	0.8–5.6	OSFED: 5.1–8.2 UFED: 1.4	16.3

NB^*^: Only ranges (not pooled data) of interview data are reported by Dahlgren et al. [27] for a more detailed comparison see Additional file 1: Table S1

*Pub.* publication, *AN* anorexia nervosa (ICD-11 Code: 6B80), *BN* bulimia nervosa (ICD-11 Code: 6B81), *BED* binge eating disorders (ICD-11 Code: 6B82), *EDNOS* eating disorder not otherwise specified (DSM-IV and earlier), *ED* eating disorders, *OSFED* other specified feeding and eating disorder (ICD-11 Code: 6B8Y), *UFED* unspecified feeding and eating disorder (ICD-11 Code: 6B8Z), *F* female, *M* male

Notwithstanding the variability in the incidence and prevalence of different EDs in the Australian and global population, it is clear that EDs have a significant effect on the health and quality of life of a wide range of individuals across all demographic categories. Evidence presented in this RR is by life stage (“children and young people” and “adults and older people”) to allow for more direct comparison between study populations. We also present evidence (or lack thereof) for specific population cohorts of interest including well-specified EDs in males, Aboriginal and Torres Strait Islander people, and among the LGBTQI + community. No studies could be identified that reported on the prevalence of well-specified EDs in the culturally and linguistically diverse (CALD) communities of Australia. It is particularly important to consider these populations, as observed longitudinal trends from 1997 to 2010 suggest that patients presenting to ED services are increasingly male and non-Caucasian [112].

Ages of participants included in studies were generally consistent across studies, including children and adolescents from the age of 11 to 19 or 20. Exceptions were two cohorts, one from Finland (Nagl et al.) where participants were aged 14 to 24, and the other from the US (Rozzell et al.) where participants were significantly younger, aged 9 and 10 [108, 118].

### Incidence and prevalence

#### Incidence

Most information on incidence is derived from clinical registries and surveillance samples and needs to be interpreted in the context of the age range of the sample. A longitudinal Swedish study (n = 286,232) reported a peak age of onset between 15 and17 years, with an incidence rate for all EDs, of 698/100,000 years in females and 55/100,000 years in males [34]. A longitudinal registry study of older adolescents in Denmark (n = 966,141) reported peak ED incidence between 16 and 20 years with a rate of 7.84/10,000 years for AN; the peak age of onset was younger in men than women for both AN (ICD-11 Code: 6B80) and BN (ICD-11 Code: 6B81) [146]. In younger children and adolescents, the peak mean age of ED onset has been reported to be between the ages of 12 and 13 years old for ARFID (ICD-11 Code: 6B83) and for the other main EDs [52, 81, 110].

In a 14-month national surveillance study of UK children ≤ 13 years old, Nicholls et al. [110] reported an incidence rate of 3.1 per 100,000 person years^1^ (PYs). Further analysis by age group found that incidence peaked between ages 12 and 13 years, with an incidence rate of 9.5 per 100,000 PY; this led the authors to conclude that mean age of ED onset may be getting younger overtime in the UK [110]. Similarly, in Canadian children (n = 2453, aged 5–12 years) the highest incidence rate of restrictive type EDs was in girls aged 10–12 years (9.4/100,000 PYs) [114]. The lowest incidence rate was in boys aged 5–9 years (0.4/100,000 person years) [114]. An Australian national paediatric surveillance sample also reported a younger age of onset as low as 8, and even 5, years old [90]. Additionally, this latter study found that approximately one-quarter of all new cases were male; furthermore, no significant differences were found between boys and girls in terms of age of onset, symptomology, family history or outcome [90].

#### Global prevalence

Table 2 summarises the prevalence estimates from the four meta-analyses and systematic reviews [9, 24, 25, 26]. It should be noted that the meta-analyses by Kessler et al. [24] and Erskine and Whiteford [26] reported on BED (ICD-11 Code: 6B82) prevalence alone and not in the context of other ED. Only one study—by Galmiche et al. [9]—provided a more thorough estimate of global ED prevalence. Studies by Kessler et al. [24] and Erskine and Whiteford [26] both used data from World Health Organization (WHO) Mental Health Surveys and analysed these data against national income categories as defined by the World Bank. While findings from Kessler et al. [24] suggest that prevalence of BN (ICD-11 Code: 6B81) and BED (ICD-11 Code: 6B82) is higher in upper-middle-income countries than in high-income and lower-middle-income countries, it is limited by the inclusion of far fewer samples from lower-middle-income countries (n = 1) and upper-middle-income countries (n = 3) compared with high-income countries (n = 10) [24].

Consistent with the findings from Kessler et al. [24] a subsequent meta-analysis conducted by Erskine and Whiteford [26] found no significant differences in prevalence of BED between high-income countries and lower-middle-income countries [24, 26]. Nonetheless, the authors did note that populations from the lower-middle-income countries included in the sample had higher obesity relative to other lower-middle-income countries, potentially contributing to the increased detection of BED (ICD-11 Code: 6B82) in these populations [26]. Reported 12-month prevalence^2^ for both genders were consistent between the Kessler et al. [24] and Erskine and Whiteford [26] meta-analyses: 0.7% and 0.8%, respectively.

A systematic review of ED prevalence (as defined by the DSM-5 in non-clinical samples) in high-income countries conducted by Dahlgren et al. [27] reported prevalence ranges for AN (ICD-11 Code: 6B80), BN (ICD-11 Code: 6B81), BED (ICD-11 Code: 6B82), OSFED (ICD-11 Code: 6B8Y) and UFED (ICD-11 Code: 6B8Z) (see Additional file 1: Table S1). Dahlgren et al. [27] noted that updates to diagnostic criteria contained in the DSM-5 resulted in an increase in individuals meeting criteria for a ‘full-threshold’ disorder. This in turn increased the prevalence of AN, BN, and BED and significantly decreased the prevalence of OSFED (previously EDNOS) [27]. Removal of the amenorrhea criterion increased diagnostic sensitivity for cases of male AN, which is also likely to have contributed to the increase in prevalence of AN following the introduction of the DSM-5 [27, 149]. It was noted that the lower limits of prevalence ranges reported in Table 2 tend to reflect studies of all-male samples while the upper limits of ranges tend to reflect all-female samples.

#### Prevalence of recently specified or reclassified disorders

##### EDNOS, OSFED and UFED

There is a much smaller evidence base for less well specified EDs compared with AN (ICD-11 Code: 6B80), BN (ICD-11 Code: 6B81), and BED (ICD-11 Code: 6B82). Table 3 shows prevalence of OSFED (ICD-11 Code: 6B8Y) and UFED (ICED-11 Code: 6B8Z) observed in all community-based studies included in this RR. Findings indicated a considerable variation across OSFED/EDNOS community prevalence studies. General population prevalence studies in adolescents (n = 9244) and adults (n = 879) conducted in the US found the lifetime prevalence of EDNOS to be 4.8% in adults and 4.6% in adolescents [89]. Even with changes to diagnostic criteria, OSFED and UFED are still common EDs.

**Table 3:** Prevalence of OSFED and UFED in community samples

	OSFED (all) (%)	S-BN (%)	S-BED (%)	A-AN (%)	UFED (%)
*Lifetime prevalence*					
Mustelin et al. [106]	F: 0.6				F: 0.9
Fairweather-Schmidt [55]	5.0				
Micali et al. [97]	F: 7.6	F: 1.4	F: 0.9	F: 1.7	F: 0.1
Mohler-kuo et al. [103] *			1.2 F: 0.9 M: 1.6		
Lahteenmaki et al. [85]		3.2 F: 6.0			
Preti et al. [115]			0.7 F: 0.6 M: 0.9		
Le Grange et al. [89]	A: 4.8 a: 4.6				
Nagl et al. [108]	G: 2.2 B: 0.7				
Isomaa et al. [76]		7.7	1.3		
*3-Month prevalence*					
Hay et al. [72]	3.2	0.5	0.4	2.5	10.4
Hay et al. [73]	8.2	0.7	6.9		1.4
*Point prevalence*					
Mitchison et al. [150]	11.2 G: 14.5 B: 8.5	2.1 G: 2.7 B: 1.2	0.3 G: 0.5 B: 0	2.9 G: 4.8 B: 1.2	6.3 G: 14.5 B: 8.5
Allen et al. [30]	G: 4.1 B: 0.9				
Flament et al. [60]	G: 6.6 B: 1.9	G: 3.7 B: 0.7		G: 1.4 B: 0.4	
Smink et al. [123]	G: 0.3 B: 0				
Rozzell et al. [118]	0.7 G: 0.7 B: 0.7	0.1 G: 0 B: 0.2	0.6 G: 0.6 B: 0.5		

*Pub.* publication, *AN* anorexia nervosa (ICD-11 Code: 6B80), *BN* bulimia nervosa (ICD-11 Code: 6B81), *BED* binge eating disorders (ICD-11 Code: 6B82), *EDNOS* eating disorder not otherwise specified, *ED* eating disorders, *OSFED* other specified feeding and eating disorder (ICD-11 Code: 6B8Y), *UFED* unspecified feeding and eating disorder (ICD-11 Code: 6B8Z), *S-BN* subthreshold (atypical) BN, *S-BED* subthreshold (atypical) binge eating disorder, *A-AN* atypical AN, *A* adults, *a* adolescents, *F* female, *M* male, *B* boys, *G* girls

##### ARFID

Little evidence was available on ARFID (ICD-11 Code: 6B83) in the general population, and prevalence is generally considered uncertain [36]. Hay et al. [72] found a 3-month prevalence of 0.3% for ARFID in the Australian population. In a Swiss study involving 1444 children aged 8 to 13, the prevalence of ARFID features was 3.2% (n = 46) [84]. However, Kurz et al. [84] noted these children may not meet full DSM-5 criteria [84]. The remaining studies assessing prevalence of ARFID were conducted within North American clinical samples (Canada and the US) and none were conducted in adults. Additional file 1: Table S2 provides a summary of prevalence rates ascertained in clinical settings.

##### Night Eating Syndrome (NES)

In contrast to other types of OSFED (ICD-11 Code: 6B8Y), the recently defined NES has a limited body of evidence relating to its prevalence. It has been found to range from 0.7% in adult men [58] and up to 4.9% in adolescent boys [150] (See Additional file 1: Table S3 for a summary of prevalence rates from the four NES studies reviewed).

##### Purging disorder

Only one study has reported lifetime prevalence of PD, which is estimated to be at 2.1% [97]. Period- and point- prevalence is reported in Table 4 and as shown PD is more prevalent among females.

**Table 4:** Prevalence of Purging Disorder (PD) in community-based samples

Author(s)	Measure	PD prev. (%)	Sample population
Micali et al. [97]	Lifetime	2.1	5658 women (aged over 40) UK community-based cohort
Mitchison et al. [150]	Point	3.2 G: 4.8 B: 1.6	5194 (ages 11 to 19) Australian school sample
Flament et al. [60]		G: 1.5 B: 0.7	3043 (ages 11 to 20) Canadian school sample
Hammerle et al. [69]		1.9	1775 (ages 13 to 14) German school sample
Hay et al. [72]	3-Month	0.3	5337 (aged 15 and over) Australian community sample
Hay et al. [73]		0.6	1476 (aged 15 and over) Australian community sample

*Prev.* Prevalence, *PD* Purging Disorder, *B* boys, *G* girls

### Sociodemographic distribution

In this section we present research with a primary focus on the prevalence of the main EDs.

#### Aboriginal and Torres Strait Islander individuals

The RR identified limited data pertinent to EDs in Aboriginal and Torres Strait Islander people [39, 71]. Within a sample of 3047 adults randomly selected to participate in a South Australian household survey, there were a total of 159 Aboriginal and Torres Strait Islander respondents. Results indicated that ED symptoms within this group, particularly rates of binge eating, were higher than in non-indigenous people (17% compared with 6.9% for non-Indigenous people) [71]. A smaller prevalence study corroborated findings that EDs were very prevalent in First Australians, and often associated with increased binge-eating frequency, lower Mental Health Related Quality of Life (MHQoL), and higher levels of overvaluation of body shape and weight compared to other Australian’s [39].

#### Children and adolescents

Due to their early age of onset, there has been considerable attention to EDs in children and adolescents. As mentioned above, there is also evidence suggesting the age of onset for EDs is getting younger [112, 151]. Data from national surveys has found that ED behaviours, including rarely studied behaviours such as chew and spit (ChSp), are widespread among Australian adolescents [150, 152]. In one study more than one-quarter (n = 628, 25.7%) of participants (aged between 13 and 17 years old) were assessed as having disordered eating, while 11% (n = 267) had a suspected ED and 0.9% had a lifetime ED [124]. The prevalence of fear of weight gain and overvaluation of body weight were also high at 14.3% to 25.7% in 3270 Australians aged 14 and 15 [75]. However, the prevalence of binge eating, and compensatory behaviours has been reported to be low (0.5% and 3.7%) [75].

Compared with adult and older populations, more comprehensive evidence exists for the prevalence of newly defined DSM-5 disorders in samples of children and adolescents. Lifetime prevalence of any ED has been estimated to be 6.7% in children and adolescents [108]. Table 5 gives a summary of point prevalence estimates from community-based samples across six studies: BN (ICD-11 Code: 6B81) is one of the most prevalent of the well-specified disorders [9, 30, 150]. A prospective longitudinal study of adolescents by Allen et al. [30] found a significant increase in ED prevalence between ages 14 (8.5%) and 17 (15.2%) and remaining steady to age 20 (15.2%). Age 17 was the peak age for all ED diagnoses (not necessarily onset), except for BED (ICD-11 Code: 6B82), which peaked at age 20 (4.1%) [30].

**Table 5:** Point prevalence of selected EDs among child and adolescent community-based samples

ED	Mitchison et al. [150] (%)	Allen et al.* [30] (%)	Hammerle et al. [69] (%)	Flament et al. [60] (%)	Smink et al. [123] (%)	Rozzell et al. [118] (%)
*AN*						
F	1.3	1.4	–	0.1	1.2	0.2
M	0	0	–	0	0.1	0
All	0.7		0.3	Not reported	Not reported	0.1
*BN*						
F	7.7	8.7	–	2.0	0.6	0
M	1.8	0.7	–	1.3	0.1	0
All	4.6		0.4	Not reported	Not reported	0
*BED*						
F	1.8	1.4	–	0.7	1.6	0.5
M	0.2	1.2	–	0.2	0.3	0.7
All	1.0		0.5	Not reported	Not reported	0.6
*Any ED*						
F	32.9	15.2	–	9.5	3.7	1.4
M	12.8	2.6	–	3.4	0.5	1.6
All	22.2	Not reported	21.0	Not reported	Not reported	1.4

^*^Data displayed for the Allen et al. [30] study was measured at mid-point (age 17)

*AN* anorexia nervosa (ICD-11 Code: 6B80), *BN* bulimia nervosa (ICD-11 Code: 6B81), *BED* binge eating disorders (ICD-11 Code: 6B82), *ED* eating disorders, *F* female, *M* male

The studies by Mitchison et al. and Allen et al. were conducted in Australian populations, with cross-sectional and longitudinal cohort designs, respectively. Mitchison et al. [150] tracked adolescents aged from 11 to 19, while Allen et al. [30] measured point prevalence at ages 14, 17 and 20. Notably, rates reported by Mitchison et al. [150] were classified as ‘probable’ and the need to apply the clinical significance criterion^3^ when assessing population-based ED prevalence was emphasised [150]. Strict application of this criterion reduced prevalence of any ED from 22.2% in the sample population to 13.6%, still considerably higher than rates reported in Canada and the Netherlands [60, 123]. Researchers argued that, without application of this criterion, ED prevalence may be overestimated in population studies for most EDs, aside from AN (ICD-11 Code: 6B80) and atypical AN. Percentages presented in Table 5 are those reported by Mitchison et al. [150] without clinical significance criteria applied, to allow for comparison with other prevalence studies. Despite the relatively high prevalence in the Australian compared to the Canadian sample (n = 3020) [60] and the Dutch sample (n = 2230) [60, 123] the Australian’s data were comparable to findings from a German study (n = 1654) [69] (see Table 5).

#### Adults and older people

##### Adults

Several studies suggest that EDs are becoming more prevalent across a range of socio-demographic profiles [9, 24, 73, 99]. Studies measuring trends in ED behaviours (as distinct from diagnosis) in the Australian population across 1995 (n = 3001), 1998 (n = 3010), 2005 (n = 3047) and 2008 (n = 3034), indicated that both binge eating and strict dieting had increased significantly in men and women, particularly binge eating in individuals > 45 years old [48, 99]. Significant increases in purging behaviours were also observed among people aged over 45 years and in males of any age [99]. Measurement of objective binge eating^4^ episodes over a 17-year period (1998 to 2015) in a large sample of Australians (n = 15,126) found 3-month prevalence increased from 2.7% (n = 80) to 13% (n = 390), and twice weekly objectively measured binge eating increased from 1.1% (n = 33) to 5.3% (n = 158) [47, 101].

Increased engagement in ED behaviours within the population could potentially translate to an increase in individuals diagnosed with EDs, especially those characterised by bingeing and purging. This is reflected in 3-month prevalence estimates of well-specified disorders in Australians aged 15 and over in two studies—Hay et al. [73] (n = 6041) and Hay et al. [72] (n = 5737) [72, 73]. Both studies used a cross-sectional design, with the earlier sample measuring ED levels in 2008 and 2009 and the later study in 2014 and 2015.

##### Middle aged and older people

Few community studies have reported EDs in populations over the age of 40 years, and even fewer in older men. This is despite findings by Ackard et al. [15] that the prevalence in people middle-aged or older has increased over time. Point prevalence studies indicate that, while EDs are less common among older individuals, they continue to be a health concern for this group [33]. Additionally, cross-sectional studies have identified individuals aged 45 to 54 as being at particular risk of experiencing ED symptomology [33].

Evidence presented in a study of women aged between 40 and 60 in Austria (n = 436) by Mangweth-Matzek et al. [92] has suggested that menopause, like puberty, may be a period of risk for ED onset [92]. Research also suggests that in older adults with EDs, comorbidities are more frequent, ED symptoms are less severe, and purging and self-harm are less frequent [37, 53, 98]. Older adults with an ED typically experienced early onset and developed a persistent ED with no period of remission [33, 97].

There are inconsistent findings in regard to how the prevalence of ED in older adults compares (i.e., is less than) to the prevalence in younger age groups [37, 53, 98]. A review of community samples by Baker and Runfola [33] found the lifetime prevalence of EDs in women aged ≥ 45 to be 0.17% for AN (ICD-11 Code: 6B80), 0.21% for BN (ICD-11 Code: 6B81), 0.61% for BED (ICD-11 Code: 6B82), and 4% for any ED. A further systematic review of EDs in people aged over 50 years found AN (ICD-11 Code: 6B80) to be the most common ED among older individuals seeking treatment, with one study reporting that AN accounted for 81% (n = 39), BN (ICD-11 Code: 6B81) in 10% (n = 5) of cases, BED (ICD-11 Code: 6B82) 2% (n = 1) and EDNOS (DSM criteria used), 6% (n = 3) [86, 103, 133, 136].

A distinctive study with a large two-phase retrospective longitudinal cohort study design involving 5658 women living in the UK, indicated that 15.3% (n = 332) had met the diagnostic criteria for an ED by the age of 40. Weighted 12-month prevalence of any ED in the cohort was 3.6% (n = 108) [97]. Prevalence rates by ED diagnosis and subtype are summarised in Table 6. This study also found the median age of onset for AN-restricting type to be 16 years (lowest), while women with BED had the highest median age of onset at 26 [97]. This finding is consistent with existing evidence that AN (ICD-11 Code: 6B80) has the youngest age of onset followed by BN (ICD-11 Code: 6B81) and then BED (ICD-11 Code: 6B82). Rates of AN in this cohort are considerably higher than in other community-based populations, while reported prevalence estimates for BED were lower [97].

**Table 6:** Lifetime prevalence of EDs detected by Micali et al. [97] in a study of 5542 women from the UK [97]

ED	ED subtype	Lifetime prevalence (%)	Number
Any ED		15.3	332
AN	All	3.6	105
	Restricting type (AN-R)	2.1	51
	Binge-Purge type (AN-BP)	1.7	54
BN	–	2.2	68
BED	–	2.0	62

*AN* anorexia nervosa (ICD-11 Code: 6B80), *BN* bulimia nervosa (ICD-11 Code: 6B81), *BED* binge eating disorders (ICD-11 Code: 6B82), *ED* eating disorders, *AN-R* AN restricting type, *AN-BP* AN binge-purge type. Source: Micali et al. [97] ‘Lifetime and 12-month prevalence of eating disorders among women in midlife: a population-based study of diagnosis and risk factors’

#### Men and LGBTQI + samples

##### Men

There is growing recognition of the impact of ED in males. It is estimated that one in four paediatric patients in Australia presenting to an ED service are male, as are one in three in the UK [7]. Few large-scale studies have focused on male prevalence in community-based populations. In their review of several Western countries, Murray et al. [7] reported community point prevalence of AN (0.1–0.3%), BN (0.1–1.6%), and BED (0.3–2.0%) in men.

##### *LGBTQI* + *communities*

Research indicates that EDs have higher prevalence in LGBTQI + individuals. EDs are more typically associated with individuals identifying as male within the LGBTQI + community, although there is growing evidence that it also has a heightened impact on females in this group [96], and there is a small volume of emerging evidence on prevalence in other genders. A systematic review suggested that greater overall ED symptomology is displayed by sexual minority males, females, and transgender individuals compared with heterosexual males and females [8]. A small study of transgender youth in Canada (n = 97) also found that risk of ED was higher among transgender males than in females, while both groups were more at risk than the general population [56].

Adolescence is a particularly risky time for ED development in LGBTQI + people [31]. A study conducted in 46 schools (n = 2429) in the US found that gay males were 12.6 times more likely to engage in fasting, vomiting or taking pills to lose weight than heterosexual males, and 2.4 times more likely to exercise or eat less to lose weight. Bisexual females were two times more likely to report fasting, vomiting or taking pills than heterosexual females, but less likely than heterosexual females to exercise or eat less to lose weight [153]. Similar trends in binge/purge behaviours among homosexual and bisexual males and females was also observed in a much larger US youth sample (n = 55,597) by Watson et al. [139]. Watson et al. [138], in a separate study (n = 26,002), found that rates of diet pill use, vomiting and fasting among lesbian females was particularly prevalent in those aged 12 to 18 [138].

In an Australian and New Zealand sample, high rates of body and muscle dysmorphia were detected among gay and bisexual men (n = 2733), who are more likely to participate in anabolic steroid use to build muscle [68]. Results from the UK (n = 5048), indicate that body dissatisfaction and dysfunctional eating behaviours in sexual minority males was up to 12.5 times higher than in heterosexual males by the age of 16 [40].

Meneguzzo et al. [96] reported that the prevalence of EDs in LGBTQI + women may be higher compared with rates reported in heterosexual women in the community. However, findings appear to be inconsistent and were not found for any particular ED diagnosis (i.e. AN, BN, or BED) [96]. Only 7 of the 45 (16%) studies included in the synthesis reported on diagnostic status [96].

### Disease burden and impact on quality of life

EDs represent a significant proportion of the global disease burden from psychiatric illnesses, with associated high levels of psychological stress and impairment, as well as a profound impact on physical health [154]. A systematic analysis of data from 195 countries from 1990 to 2017 found that the global disease burden for any ED was 43.4 age-standardised disability adjusted life years (DALYs)^5^ per 100,000. Between 2007 and 2017, global disease burden caused by EDs increased by 9.4%. AN (ICD-11 Code: 6B80) and BN (ICD-11 Code: 6B81) were the only EDs initially specified by the Global Burden of Disease Study 2017, at 9.5 and 33.8 age-standardised-DALYs per 100,000, respectively. Global disease burden attributed to AN (ICD-11 Code: 6B80) increased by 6.1% between 2007 and 2017, and for BN (ICD-11 Code: 6B81) the burden increased by 10.3% [155]. This burden further doubled when BED (ICD-11 Code: 6B82) and OSFED (ICD-11 Code: 6B8Y) were counted as part of measuring burden (disability life adjusted years), in part due to the recognition of BED and OSFED in a large global study of burden of disease [120].

Erskine et al. [54], in their review of the 2013 Global Burden of Disease Study, highlight that much of the disease burden associated with EDs is experienced by females, with reported age-standardised-DALYs due to all EDs being over twice as high for females than for males [54]. In AN, the difference was even more pronounced at over four times higher in females [155].

In Australian populations, investigation of disease burden attributable to more recently specified DSM-5 disorders indicated that individuals with BN (ICD-11 Code: 6B81) and ARFID (ICD-11 Code: 6B83) had more days out-of-role^6^ than individuals without an ED and for other ED diagnoses [72]. Further, engaging in binge eating behaviours while not necessarily being diagnosed with an ED was also found to have an impact on daily functioning for Australians (n = 15,126). Mitchison et al. [101] found that participants who reported once or twice weekly objectively measured episodes of binge eating had higher role impairment than individuals who did not report objective binge eating. Observing 18-year trends, Mitchison et al. [101] also reported marked increases in binge eating within the Australian general population, potentially contributing to increased weight and poor physical health over time.

#### Economic impact

Two studies assessing the economic burden of EDs were identified. Agh et al. reviewed 22 studies relating to healthcare costs and economic burden associated with AN (ICD-11 Code: 6B80), BN (ICD-11 Code: 6B81) and BED (ICD-11 Code: 6B82) [156]. They found that, while individuals with BED had a higher rate of service utilisation, including inpatient, outpatient and emergency care than healthy controls, levels were comparable to individuals with other psychiatric disorders. It was also noted that very few individuals sought help specifically for their ED, but did so for comorbid psychiatric conditions or for assistance with weight loss [156]. Agh et al. reviewed the cost of services such as therapy, hospital care, diagnostic tests, and medications accessed by ED patients in the US (n = 14), the UK (n = 1), Canada (n = 1) and Germany (n = 5), including studies that measured costs from the perspective of the payer (consumer) (n = 15), hospital/health service (n = 3) or society (n = 3). A diagnosis of AN was associated with highest healthcare costs and longer periods of hospitalisation compared to other well-specified EDs [156]. Estimated annual healthcare costs were reported in Euro (€) and converted to AUD ($) for EDs. Data from the analysis by Agh et al. indicated that the high costs associated with AN were due to longer periods of hospitalisation [156].

A recent study from the general population of South Australia estimated the total economic cost of all EDs was $AUD84 billion in 2018, from years of life lost due to disability and death, and annual loss of earnings accounted for $AUD1.646 billion. Furthermore, these lost earnings peaked for both males and females in the age group 35–44 years [130].

#### Quality of life impact

Individuals with EDs have been found to have lower Health Related Quality of Life (HRQoL) than the general population and individuals with other psychiatric disorders such as major depression [141]. Research on the impact of ED behaviours indicated that HRQoL was equally impacted by a range of ED types, including binge eating, strict dieting, and purging. Distress relating to binge eating was associated with greater functional impairment and lower QoL in trends tracked from 1998 to 2015 in the Australian population [99]. Among school-aged children in Austria (n = 3610), poorer HRQoL was found among females at high risk of ED, potentially indicating more severe symptomology in female adolescents [144].

A meta-analysis of seven studies conducted by Winkler et al. [141] comparing HRQoL between AN (ICD-11 Code: 6B80), BN (ICD-11 Code: 6B81), BED (ICD-11 Code: 6B82) and EDNOS found equally low HRQoL scores across all diagnostic groups with no significant differences between groups [141]. However, researchers noted that this finding was from a limited pool of studies that use a range of HRQoL measures both specific to ED (Eating Disorder Quality of Life, EDQoL) and generic measures [141].

Extremely low BMI experienced by individuals with AN (ICD-11 Code: 6B80) is considered to have a substantial impact on their physical health. However, the egosyntonicity of symptoms may result in lower-than-expected levels of reported mental health impact. In contrast symptoms of BN (ICD-11 Code: 6B81) and BED (ICD-11 Code: 6B82) are experienced with high levels of associated psychological distress, hence individuals with BN and BED have been found to have lower HRQoL than individuals with AN [32, 93, 141].

In studies conducted in the Australian population, BN (ICD-11 Code: 6B81), BED (ICD-11 Code: 6B82), and ARFID (ICD-11 Code: 6B83) were associated with lower HRQoL (particularly lower mental health quality of life, MHQoL) compared with other ED diagnoses and individuals without ED. Australians with BED (ICD-11 Code: 6B82) were found to score lower than individuals with AN (ICD-11 Code: 6B80), BN (ICD-11 Code: 6B81), OSFED (ICD-11 Code: 6B8Y) and UFED (ICD-11 Code: 6B8Z) for mental and physical HRQoL [72]. Compared with healthy Australian women (n = 232), a much higher proportion of women with EDs (n = 159) were assessed to have severe mental health impairment; at 29.8% versus 9.4% [104]. Similar impairments to physical and mental HRQoL were observed among a sample of women in New Zealand (n = 214) with more frequent binge eating associated with lower QoL [134]. Further, longitudinal observation of ED status and HRQoL in Australian women (n = 706) indicated a bi-directional relationship, whereby increasing ED symptomology leads to greater QoL impairment and conversely lower QoL contributes to ED severity over time [100].

Several studies have also reported poor HRQoL for ARFID (ICD-11 Code: 6B83) in young people (e.g. Krom et al. [83] and adults [72]). Krom et al. [83] found that patients aged 6 to 7 and 8 to 10 years with ARFID (n = 48), had significantly lower physical functioning (appetite, lungs, stomach and motor) and mental health (positive mood and liveliness). Psychosocial health and school functioning measures were also significantly lower in this group indicating that ARFID has a significant negative impact on QoL [83].

## Discussion

This RR presents a contemporary understanding of the epidemiology of EDs, their sociodemographic distribution, particularly across age and gender, and their comorbidity and burden. It guides the AEDRTS and policy as well as informing the field and Stakeholders more broadly.

### Prevalence and incidence

Collectively, epidemiological evidence form this RR suggests that the incidence of EDs is increasing, while age of onset is decreasing. However, as incidence estimates come mainly from studies using registry or clinical data, they are likely underestimates as they only include cases that have been formally diagnosed by a health professional. For example, reported rates in the UK were considerably higher than incidence reported in Australian children aged between 5 and 13 [110]. This variance may be due to differences in methodologies, as some Australian studies, such as that by Madden et al. [90], were predominantly reported from inpatient services with only a small proportion of outpatient services.

The RR found that EDs are a global and common phenomenon but only one meta-analysis [9] provided a comprehensive synthesis of epidemiology for all EDs, and there is a paucity of evidence regarding more recently specified disorders such as ARFID (ICD-11 Code: 6B83). Prevalence rates (Table 2) also varied considerably between studies most probably due to differences in study design and measures used to detect EDs. Treasure et al. [132, 157] argue that AN (ICD-11 Code: 6B80) prevalence is impacted by inconsistent use of strictly defined parameters relating to body mass index (BMI) limits, contributing to the variation. This was demonstrated by application of broad and strictly defined parameters for AN (ICD-11 Code: 6B80) to the same community-based samples of female adolescents. Application of strictly defined AN (ICD-11 Code: 6B80) parameters resulted in an observed lifetime prevalence range between 0.6 and 2.2%. However, using broadly defined AN (ICD-11 Code: 6B80) parameters in the same sample, ranges for lifetime prevalence increased to 1.7% to 4.3% [132].

With regards to prevalence for OSFED (ICD-11 Code: 6B8Y) disorders it is important to note that these appeared to be hierarchical in nature [9, 25, 27, 54]. That is, only one diagnosis assigned at a time, despite potential for overlap. Thus, individuals who were diagnosed with OSFED-PD (purging in the absence of binging) could also have met DSM-5 criteria for atypical AN. It should be noted also that some studies (such as Micali et al. [97]) did not specify purging for the purpose of weight and shape concerns. However, considering the measures used (EDDS, SCID-I, LIFE) it may be assumed that PD was derived in the context of EDs, where weight and shape concerns are present. The exact diagnostic boundaries between EDNOS/OSFED/UFED are often difficult to delineate, or diagnose, in non-clinical samples as it is dependent on how researchers define these broad categories, particularly as both the DSM and ICD do not outline strict criteria for these diagnostic categories.

Increases in BN (ICD-11 Code: 6B81) and BED (ICD-11 Code: 6B82) prevalence [73] over time could be attributed to the broader DSM-5 criteria, which reduced the number of required binge eating episodes from twice weekly in the DSM-IV to once weekly. Similarly, changes in the AN (ICD-11 Code: 6B80) diagnostic criteria to remove amenorrhea and vary the weight cut-off likely also play a role in rising prevalence data. Strict diagnostic criteria specified by the DSM-IV decreased BN (ICD-11 Code: 6B81) cases by half and BED (ICD-11 Code: 6B82) cases to less than half, bringing 3-month prevalence down to the same rate detected in the earlier 2005 South Australian study using once weekly criteria [73, 158]. This finding suggests that prevalence rates of BN (ICD-11 Code: 6B81) and BED (ICD-11 Code: 6B82) in the Australian population in 2005 were comparable to rates reported in the 2015 study but not detected using DSM-IV criteria. Further analysis of prevalence rates by participant characteristics found several key differences between studies from 2005 to 2015. In 2015 studies, the median age of participants with an ED was significantly younger than the group without an ED, particularly in AN (ICD-11 Code: 6B80) and BN (ICD-11 Code: 6B81). Further, BED (ICD-11 Code: 6B8) (57% female) and subthreshold BED (S-BED; 55% female) had the lowest sex (female-to-male) ratio of all reported EDs, and BED, S-BED and BN were found to be associated with high BMI [73].

### Prevalence: child and adolescence

Reported lifetime and point prevalence rates varied considerably across studies [76, 108, 129]. However, despite limitations across included studies, literature indicates that less well-specified EDs may be more prevalent in children and adolescents than adult populations [30, 60, 75, 123, 124, 150]. Whilst the prevalence of well-specified EDs (AN, BN and BED) in Australian female adolescents was generally consistent, conflicting prevalence rates were observed in studies of males for BN (1.8% [150] compared to 0.7% [30]), and BED (0.2% [150] compared to 1.2% [30]).

### Prevalence: males and LGBTQI +

The literature notes that males may preference different body types than females, typically presenting with higher BMIs and a drive for muscularity instead of thinness (muscle dysmorphia versus body dysmorphia), as well as reporting less psychological distress relating to binge eating behaviours [7]. These characteristics are more commonly associated with BN (ICD-11 Code: 6B81) and BED (ICD-11 Code: 6B82) and may reflect the relatively low prevalence of AN (ICD-11 Code: 6B80) in males [65, 115]. Males are also more likely to report overeating without loss of control while eating, commonly reported by females, resulting in a higher proportion of males with S-BED [103, 115] and a higher proportion of females diagnosed with full syndrome BED [127]. It may be that binge eating presents differently in males and this warrants further investigation to ensure diagnostic criteria do not contain inherent bias and lead to an inaccurate estimation of prevalence.

Researchers have also observed that changes to diagnostic criteria from DSM-IV to DSM-5 resulted in apparent increases in the prevalence of EDs in females, although male prevalence was largely unchanged for the more common threshold and subthreshold EDs (e.g., AN, S-AN, S-BN). This may indicate that the diagnostic criteria remain largely female-centric even though ED symptomology and behaviours are relatively common among males [7, 43]. For example, Compte et al. [43] observed no difference in prevalence rates comparing DSM-IV and DSM-5 diagnosed EDs in a group of university-aged men (n = 472). All observed cases in males were subthreshold AN (S-AN) (0.9%, n = 4) and subthreshold BN (S-BN) (1.1%, n = 5) [43]. However, in the same sample group, muscle dysmorphia was determined to occur in 7.0% of men [43], representing more than a six-fold increase in prevalence of eating pathology compared to other presentations in this illness category. This supports additional academic and clinical focus on ED in males given that clinical data has demonstrated that EDs have a considerable impact on males [121], accounting for 34% of all patients accessing ED services in one study [121].

There is a dearth of consistent epidemiological data on EDs in the LGBTQI + community. However, the evidence reviewed here suggests they may be a particularly vulnerable minority group for EDs and further research is needed [96].

### Economic impact

This RR found high fiscal burden from EDs. For example, in a study modelling the cost-effectiveness of an AN prevention, the annual cost of treating an individual with AN (ICD-11 Code: 6B80) was estimated at up to $USD200,000 [130]. This review however found variation in these costs across different countries; in Australia some data suggests a higher cost for BN (ICD-11 Code: 6B81) relative to AN (ICD-11 Code: 6B80) whereas in the US it is reversed, which may be related to the differences in health systems across the two countries. The high costs of care for individuals with AN (ICD-11 Code: 6B80) are associated with lengthy hospital stays, which in Australia are often partially or completed publicly funded, whereas in the US (where a significant proportion of studies have been conducted) hospital costs tend to be paid for by the individual receiving care or under their personal insurance [156, 159].

### Quality of life

Disordered eating behaviour in general, and EDs in particular, have been consistently found to impact HRQoL in a variety of ways, in both young people and adults [10, 72, 73, 79, 141, 152, 160]. Variance in findings across diagnostic groups should be addressed in future research by using specific EDQoL measures [32, 141], as generic measures and assessment tools are inefficient at detecting the unique features that impact QoL in EDs [79]. The insensitivity of self-report HRQoL measures to the egosyntonic nature of AN (ICD-11 Code: 6B80) has also been suggested as a possible reason for conflicting findings [79, 141]—people with ED report different sorts of impacts of illness and often do not experience the traditional sort of impacts or fail to find them as distressing. Despite this, a number of studies have found a lower HRQoL in AN (ICD-11 Code: 6B80) as compared to BN (ICD-11 Code: 6B8) and EDNOS (OSFED), noting that individuals with AN (regardless of subtype or age [140]) experience greater difficulty with social life, relationships and physical mobility [79] and recognising the close association between AN (ICD-11 Code: 6B80) and suicidality. While outside the date of current review’s eligibility criteria [10] it should also be mentioned that a very recent study by Appolinario et al. [161] reported diverse and severe physical health impacts of BN (ICD-11 Code: 6B81) and BED (ICD-11 Code: 6B82), even when controlling for participants’ BMI. This corroborates similar findings of medical comorbidity in individuals with BED as noted by Udo and Grilo [162], albeit, they did not control for BMI.

### Strength and limitations of included studies

A limitation of this RR is that the vast majority of the available epidemiological literature came from Western, educated, industrialized, rich, and democratic (WEIRD) countries, which more readily have access to specialised care and services. Further, much of the evidence base for the ED literature is restricted to younger and female-only samples [9, 24, 26]. Other limitations include wide variability in methods to ascertain ED cases, including application of different diagnostic criteria and use of self-report versus interview instruments. Nonetheless, the breadth of netted literature that met inclusion criteria provided a comprehensive overview of the topic and allowed for trends and themes to be observed, highlighting both trends and gaps in the epidemiological understanding of EDs.

### Strengths and limitations of current review

Use of a rapid review methodology allowed for a timely synthesis of the current evidence base as it relates to the epidemiology of EDs. Nonetheless, as the RR was commissioned by the Australian Government to inform the focus of EDs in Australia, it did not address indigenous population in other countries. Similarly, more recently specified disorders (such as ARFID (ICD-11 Code: 6B83) and OSFED (ICD-11 Code: 6B8Y)) were not equally represented when compared to other established ED diagnoses, namely AN (ICD-11 Code: 6B80), BN (ICD-11 Code: 6B81), and BED (ICD-11 Code: 6B82). Representation of countries outside of Australia may have also impacted findings, however this was partially offset by predominantly focusing on WEIRD countries with similar sociodemographic features as Australia—allowing for some findings to be generalised.

### Overall clinical implications

EDs are common, and likely increasing in incidence and prevalence in both younger and older populations. They occur across all sociodemographic groups and may be increasing in minority populations. Thus, all services at all levels need to be prepared to identify and offer care for people with EDs. There is a need to develop culturally informed and appropriate assessments and interventions for broader demographic groups, such as men, the LGBTIQ + community and Indigenous peoples.

### Future research

An Australian nationally representative epidemiologic survey as well as research in economically developing nations, gender and culturally and linguistically populations are needed. There is also a need for greater use of a two-stage design and interview approach in prevalence studies, to increase accurate case identification and inclusion of OSFED (ICD-11 Code: 6B8Y) and UFED (ICD-11 Code: 6B8Z) in study methods. That also includes measurement of burden to improve the Global Burden of Disease (WHO) and other estimates, particularly those used in policy making around health, and the provision of care.

## Conclusion

EDs are common, global, present in all age and gender groups and are associated with high fiscal and health burden. There is an urgent need to refine and harmonise epidemiological methods to improve consistency and accuracy in case estimates, for example the development of international agreements on assessment instruments amongst eating disorder organisations and publications. Publication policies can also be implemented to ensure all papers consider and present data regarding demographic diversity of participants to support greater research in minority populations and non-WEIRD populations. Such strategies would enable a better understanding of the distribution of EDs over time, plan services and guide health care policy.

## Supplementary Information

**Additional file 1.** Prevalence rates from select epidemiological studies - includes Night Eating Syndrome and Avoidant/Restrictive Food Intake Disorder.

## Data Availability

Not applicable—all citations provided.

## Abbreviations

AEDRTS: Australian Eating Disorder Research and Translation Strategy
AN: Anorexia Nervosa
ARFID: Avoidant Restrictive Food Intake Disorder
BED: Binge Eating Disorder (BED)
BMI: Body Mass Index
BN: Bulimia Nervosa (BN)
CALD: Culturally and Linguistically Diverse
DALY: Disability Adjusted Life Years
DSM-5: Diagnostic and Statistical Manual of Mental Disorders—fifth edition
ED: Eating Disorder
EDNOS: Eating Disorder not Otherwise Specified
EDQoL: Eating Disorder Quality of Life
HMA: Health Management Australia
HRQoL: Health Related Quality of Life
ICD: International Classification of Diseases
IOI: InsideOut Institute
LGBTQI +: Lesbian, Gay, Bisexual, Transgender, Queer, Intersex +
MHQoL: Mental Health Quality of Life
NES: Night Eating Syndrome
OSFED: Other Specified Feeding and Eating Disorders
PD: Purging Disorder
PRISMA: Preferred Reporting Items for Systematic Reviews and Meta-Analyses
PY: Person Years
QoL: Quality of Life
RR: Rapid Review
UFED: Unspecified Feeding and Eating Disorders
WEIRD: Western, Educated, Industrialized, Rich and Democratic (countries)
WHO: World Health Organisation

## Footnotes

1 Person years are a unit of measurement that considers the length of time (e.g., 1 year) and number of individuals enrolled in a study. For example, 10 individuals enrolled in a 10-year study would equate to 100 person years.

2 *Proportion of a population who have had an ED in the past 12 months.*

3 *ED symptoms that cause clinically significant distress or impairment in social, occupational, or other important areas of functioning.*

4 *Sense of loss of control overeating within a specific timeframe during which the amount of food consumed is larger than what most people would eat under similar circumstances.*

5 *Number of potential ‘healthy’ years of life lost to premature death or years lived with disability due a specific disease or disorder.*

6 *Days for which a person is completely unable to work or carry out normal activities because of a health problem.*
