# Supplementary material for: Epidemiology of eating disorders: population, prevalence, disease burden and quality of life informing public policy in Australia—a rapid review
Source: J Eat Disord. 2023 Feb 15;11:23. doi: 10.1186/s40337-023-00738-7 (PMC9933292; doi:10.1186/s40337-023-00738-7)
Supplement: Supplementary file 1 — Additional file 1. Prevalence rates from select epidemiological studies - includes Night Eating Syndrome and Avoidant/Restrictive Food Intake Disorder. [file 40337_2023_738_MOESM1_ESM.docx]

# **Additional file 1**

***Table S1:***

ED prevalence ranges of studies reviewed by Dahlgren et al. (2017)

| **ED** | **Prevalence measure** | **2 stage design** | **Interview** | **Self-report** |
| --- | --- | --- | --- | --- |
| *AN* | Lifetime | 0.1%–3.6% | 0.1%–1.4% | No data |
|  | Point | 0–1.2% | 0.5%–2.0% | 0–0.6% |
| *BN* | Lifetime | 0.1%–0.8% | 2.6% | No data |
|  | Point | 0.1%–0.8% | 0.7%–1.0% | 0.4%–7.9% |
| *BED* | Lifetime | 0.7%– 2.3% | 2.7%–3.6% | 1.4%–2.6% |
|  | Point | 0.3%–3.6% | 0.8%–5.6% | 0.2%–3.6% |
| *OSFED* | Lifetime | 0.3%–0.6% | 3.8%–11.5% | No data |
|  | Point | 0.3%–2.4% | 5.1%–8.2% | 0.7%–5.5% |
| *UFED* | Lifetime | 0.2%–0.9% | No data | No data |
|  | Point | 0 | 1.4% | No data |
| *ANY ED* | Lifetime | 1.2%–5.7% | No data | No data |
|  | Point | 0.5%–7.4% | 16.3% | 2.9%–15.2% |
| ***COMBINED SAMPLE POPULATION*** | | 9,168  (5 studies) | 21,392  (6 studies) | 35,595  (8 studies) |

*Abbreviations:* AN = anorexia nervosa, BN = bulimia nervosa, BED = binge eating disorders, EDNOS = eating disorder not otherwise specified, ED = eating disorders, OSFED = other specified feeding and eating disorder, UFED = unspecified feeding and eating disorder.

***Table S2:***

Prevalence of ARFID in clinical child and adolescent samples

|  | **ARFID prev.** | **Clinical setting** | **Sample Pop.** |
| --- | --- | --- | --- |
| *Eddy et al. (2014)* | - 1.5% - 2.4% ‘possible’ | Paediatric gastroenterology patients | 2,231 children (aged 8 to 18) |
| *Fisher et al. (2014)* | - 13.8% | ED program users | 712 children and adolescents |
| *Cooney et al. (2018)* | - 8.4% | Paediatric hospital inpatients | 369 children |
| *Nicely et al. (2014)* | - 22.5% | ED service outpatients | 173 children (aged 7 to 17) |
| *Abbreviations*: ARFID = Avoidant/Restrictive Food Intake Disorder, Prev. = Prevalence, Pop. = Population, Prev. = Prevalence. *NB1*: In three out of four of the studies displayed in table above, ARFID was more common among male patients than female patients. In the sample from the Eddy et al. study, male paediatric patients (aged 8 to 18) accounted for two-thirds of observed ARFID cases, although researchers considered overall prevalence to be lower than expected in this high-risk group. This raised questions around the sensitivity of diagnostic criteria used for this study [94]. *NB2*: Individuals with ARFID were also found to be, on average, younger than patients with other EDs [95] [96]. [97]. While preliminary data from clinical samples indicates that ARFID is more common in males than females, there is a lack of community-based data on the prevalence of ARFID [67] [98]. Therefore, findings regarding gender differences remain inconclusive. | | | |

***Table S3:***

Point prevalence of NES in community-based samples

|  | **NES** | **Sample population** |
| --- | --- | --- |
| *De Zwaan et al. (2014)* | - 1.1% (n=27) | - 2,508 (ages 14 to 92) - German nationally representative |
| *Runfola et al. (2014)* | - 2.9% (n=67) | - 1,636 (aged 18 to 26) - 10 US universities |
| *Fischer et al. (2012)* | - **F:** 1.1% - **M:** 0.7% | - 1,514 (aged 18 to 26) - Swiss community sample |
| *Tholin et al. (2009)* | - **F:** 1.7% - **M:** 2.5% | - 21,741 (ages 20 to 47) - Swedish twins |
| *Mitchison et al. (2019)* | - 4.1% - **G:** 3.6% - **B:** 4.9% | - 5,194 (ages 11 to 19) - Australian school sample |

*Abbreviations*: NES = Night Eating Syndrome, F = Female, M = Male, G = Girls, B = Boys
